# The efficacy and safety of darolutamide combination therapy in advanced prostate cancer: a systematic review and meta-analysis of randomized controlled trials

**DOI:** 10.3389/fphar.2026.1818807

**Published:** 2026-05-18

**Authors:** Xi Cao, Di Li, Zhuoyi Han, Zhenghao Li, Junhao Chu, Changze Song, Hongyin Huang, Huisheng Yuan, Zilong Wang

**Affiliations:** 1 Department of Urology, Kidney and Urology Center, Scientific Research Center, Pelvic Floor Disorders Center, The Seventh Affiliated Hospital, Sun Yat-sen University, Shenzhen, Guangdong, China; 2 School of Medicine, Sun Yat-sen University, Shenzhen, Guangdong, China; 3 Department of Andrology, The Seventh Affiliated Hospital, Sun Yat-sen University, Shenzhen, Guangdong, China; 4 Department of Burns and Plastic Surgery, The Seventh Affiliated Hospital, Sun Yat-sen University, Shenzhen, Guangdong, China

**Keywords:** darolutamide, meta-analysis, metastasis-free survival, overall survival, prostate cancer

## Abstract

**Background:**

Darolutamide is the next-generation androgen receptor inhibitor approved for the treatment of advanced prostate cancer, including non-metastatic castration-resistant prostate cancer (nmCRPC) and metastatic hormone-sensitive prostate cancer (mHSPC). However, the efficacy and safety of darolutamide are nonetheless worthy of further clinical studies. The objective of this meta-analysis was to evaluate the overall survival, metastasis-free survival and various specific adverse events of darolutamide combination therapy in patients with mHSPC or nmCRPC.

**Methods:**

This meta-analysis was performed on PubMed, EMBASE, Web of Science, ClinicalTrials.gov, and the Cochrane Library for English-language articles to collect randomized clinical trials of darolutamide combination therapy in mHSPC and nmCRPC from the start of the database to 15 January 2026. The primary efficacy outcomes were overall survival and metastasis-free survival. Key safety outcomes included the total number of overall adverse events, the total number of grade ≥3 adverse events and serious adverse events, and the occurrence of specific adverse events of interest. The risk of bias was assessed by the Cochrane risk-of-bias tool for randomized trials (RoB 2). Publication bias was assessed by funnel plots.

**Results:**

There were 8 research articles from 3 randomized clinical trials with 3,483 patients involved in this meta-analysis, including 1,509 nmCRPC patients from the ARAMIS trial, 1974 mHSPC patients from the ARASENS and ARANOTE trials. Combining darolutamide with ADT significantly prolonged overall survival (OS) and metastasis-free survival (MFS) in nmCRPC patients compared with placebo plus ADT. And darolutamide plus ADT with or without docetaxel also showed favorable overall survival in mHSPC patients. Subgroup meta-analyses of OS among mHSPC patients for baseline total PSA (tPSA) values and Gleason scores showed the beneficial efficacy of darolutamide in mHSPC. The addition of darolutamide in ADT and/or docetaxel did not lead to serious adverse events, like heart failure, bone fracture and hypertension, in both nmCRPC and mHSPC patients.

**Conclusion:**

Darolutamide combination therapy was beneficial to the prognosis and demonstrated a favorable safety profile in patients with advanced prostate cancer.

**Systematic Review Registration:**

Identifier CRD420251145736.

## 1 Introduction

Advanced prostate cancer, with androgen deprivation therapy (ADT) by medical or surgical castration as the mainstay of treatment, is categorized into non-metastatic castration-resistant prostate cancer (nmCRPC), metastatic hormone-sensitive prostate cancer (mHSPC), and metastatic castration-resistant prostate cancer (mCRPC) based on sensitivity to ADT and the occurrence of distant metastasis (Scher et al., 2016). Although patients initially robustly respond to first-line ADT, nearly all with advanced prostate cancer eventually progress to lethal CRPC (Luo et al., 2018). Among them, those in whom traditional imaging examinations did not detect metastatic lesions were classified as having nmCRPC, with the median overall survival period of nearly 5 years (Heiss et al., 2024). Additionally, approximately 10% of prostate cancer patients are newly diagnosed with metastatic disease at the initial diagnosis as mHSPC, with only a 37% survival rate over 5 years (Raychaudhuri et al., 2025). Thus, the effective therapeutic strategies are urgently needed for advanced prostate cancer.

Enzalutamide, apalutamide and darolutamide are the FDA-approved second-generation AR antagonists for advanced prostate cancer treatment (Spratt et al., 2025). These novel androgen receptor (AR) antagonists have demonstrated superior prognosis in advanced prostate cancer compared with bicalutamide (Vaishampayan et al., 2021). However, resistance inevitably develops, primarily mediated by mechanisms of AR reactivation or AR-independent pathways, including glucocorticoid receptor upregulation, neuroendocrine differentiation, and activation of the PI3K/AKT pathway (Bleve et al., 2025). Conventional AR antagonists such as enzalutamide and apalutamide can be converted from antagonists to agonists in the presence of the AR (F876L) mutation. In contrast, darolutamide and its main active metabolite, ORM-15341, retain full antagonistic activity against this and other common resistance-associated mutations including AR (W741L) and AR (T877A), without converting to agonists, representing one of the mechanisms by which darolutamide overcomes resistance (Wu et al., 2022; Moilanen et al., 2015). Furthermore, darolutamide exhibits minimal interference with the CYP enzyme system, reducing the risk of drug-drug interactions. Compared with enzalutamide and apalutamide, darolutamide exhibits an extremely low brain-to-plasma concentration ratio, conferring a distinct safety advantage with respect to central nervous system tolerability (Wenzel et al., 2022). Therefore, a comprehensive systematic review and meta-analysis evaluating the efficacy and safety of darolutamide is highly warranted.

Several multicenter randomized clinical trials have focused on the efficacy and safety of darolutamide in recent years. For nmCRPC, Fizazi et al. initially announced that darolutamide has improved the median metastasis-free survival (MFS) by nearly 2 years and has reduced the risk of death by 31% compared with placebo in the phase three Androgen Receptor Antagonizing Agent for Metastasis-free Survival (ARAMIS) trial in 2019 (Fizazi et al., 2019). In terms of mHSPC, Smith et al. showed that the addition of darolutamide to ADT and docetaxel significantly increased overall survival (OS) and reduced the risk of death by 32.5% in the phase three ODM-201 in Addition to Standard ADT and Docetaxel in Metastatic Castration Sensitive Prostate Cancer (ARASENS) trial in 2022 (Smith et al., 2022). Since then, there have been a great deal of phase three randomized clinical trials on the efficacy and safety of darolutamide combination therapy in nmCRPC or mHSPC.

In this study, we conducted a meta-analysis of each randomized clinical trials up to 15 January 2026 to evaluate the efficacy and safety of darolutamide and ADT with/without docetaxel in mHSPC patients and use a narrative synthesis for nmCRPC patients. This meta-analysis will provide support for the future development of darolutamide combination therapy for the treatment of advanced prostate cancer.

## 2 Materials and methods

### 2.1 Study retrieval

Before conducting this systematic review, we registered the review protocol in the International Prospective Register of Systematic Reviews (PROSPERO) public database (CRD420251145736). A literature search was performed following Preferred Reporting Items for Systematic Reviews and Meta-Analyses (PRISMA) Guidelines. We searched PubMed, EMBASE, Web of Science, ClinicalTrials.gov, and the Cochrane Library for English-language articles to collect randomized clinical trials of darolutamide and ADT therapy with/without docetaxel in patients with mHSPC or nmCRPC, from the start of the database to 15 January 2026. Search terms included “darolutamide”, “prostate cancer” and “Randomized controlled trial”. Results were input into an EndNote library, and duplicates were removed automatically. Two independent researchers (XC, DL) screened the search results and studies with the following criteria were selected: (1) randomized control trial, (2) including patients with mHSPC and nmCRPC in the study, and (3) providing at least one of the following oncologic outcomes: overall survival (OS), metastasis-free survival (MFS) and adverse events. Any conflicts between the two reviewers were resolved by a third reviewer (ZYH). Additionally, all references of included studies were manually scanned to search for relevant articles in our study. Please refer to the Supplementary Table S1 for the detailed search strategy.

### 2.2 Inclusion criterion

Study selection and eligibility screening were conducted according to the patient population, intervention or exposure, comparator, outcome, and study design (PICOS). All patients had a clinical examination and biopsy histopathological analysis confirming advanced prostate cancer, including metastatic hormone-sensitive prostate cancer (mHSPC) or non-metastatic castration-resistant prostate cancer (nmCRPC) (P). All of these patients received treatments containing darolutamide, either doublet therapy (darolutamide plus androgen deprivation therapy [ADT]) or in combination with other agents (e.g., darolutamide plus ADT and docetaxel) (I). We compared the darolutamide group versus the placebo group, including both doublet (e.g., darolutamide + ADT) and triplet (e.g., darolutamide + docetaxel + ADT) therapy regimens (C). The primary efficacy outcomes were OS and MFS. Key safety outcomes included the total number of overall adverse events, the total number of grade ≥3 adverse events and serious adverse events, and the occurrence of specific adverse events of interest (O). Phase III randomized controlled trials (RCTs) and related subgroup analyses published as full-text articles were included (S). The review articles and case reports were excluded from our study.

### 2.3 Data extraction

Three investigators (XC, DL and ZYH) independently conducted the screen and data extraction for the literature search, with discrepancies resolved by other reviewers. Articles were first screened based on titles and abstracts, and then selected by full-text articles according to eligibility criteria. The following data were extracted for our study: authors, publication year, country, study type, quality assessment level, disease subtype, total number of cases, efficacy endpoints (including overall survival and metastasis-free survival), and safety outcomes (including total number of adverse events, severity grades, and specific events of interest). The primary outcomes of interest included overall survival (OS) and metastasis-free survival (MFS). OS is defined as time from randomisation to date of death from any cause, and MFS is defined as time from randomization to confirmed evidence of metastasis or death from any cause, whichever occurs first (Fizazi et al., 2019). The adverse event refers to any untoward medical occurrence in a study subject that does not necessarily have a causal relationship with this treatment (Fizazi et al., 2019), while a serious adverse event is any health-related event at any dose that causes death, hospitalization, disability, or congenital anomaly requiring intervention. Safety outcomes following darolutamide combination therapy were systematically evaluated based on the eight included articles (Fizazi et al., 2019; Smith et al., 2022; Saad et al., 2024; Hussain et al., 2023; Shore et al., 2024; Fizazi et al., 2020; Uemura et al., 2021; Shore et al., 2022), utilizing narrative synthesis for the nmCRPC cohort and meta-analysis for the mHSPC cohort. All adverse events in all studies were graded with National Cancer Institute Common Terminology Criteria for Adverse Events (NCI CTCAE) (Fizazi et al., 2019).

### 2.4 Quality assessment

Three investigators (XC, DL and ZYH) independently completed the quality assessment and risk of bias for all included studies. The risk of bias at the trial level was assessed using the revised Cochrane risk-of-bias tool for randomized trials (RoB 2). This tool assesses five domains (I) bias arising from the randomization process (II) bias due to deviations from intended interventions (III) bias due to missing outcome data (IV) bias in measurement of the outcome, and (V) bias in selection of the reported result. Each domain was rated as low risk, unclear risk, or high risk of bias. The overall ROB for each trial was determined based on the highest risk in any domain. Publication bias was assessed by funnel plots. Additionally, the grading of recommendations, assessment, development, and evaluation (GRADE) system (https://gdt.gradepro.org/app/) was used to evaluate the quality of the evidence. The evaluation included the study design, risk of bias, inconsistency, indirectness, imprecision, and publication bias. Because all included studies were randomized controlled trials, the initial certainty of evidence was rated as ‘high’. The evidence was then systematically downgraded based on limitations in the aforementioned domains, and other considerations and four quality levels were developed: ‘very low’, ‘low’, ‘moderate’, and ‘high’.

### 2.5 Statistical analysis

Meta-analysis was conducted using Review Manager (RevMan, version 5.4, The Cochrane Collaboration) exclusively for the mHSPC cohort. Data from the nmCRPC cohort were not pooled because multiple reports come from the single ARAMIS trial. Instead, a narrative synthesis was performed using several summary tables. For prognostic outcomes, the pooled effect sizes were expressed as hazard ratios (HRs) using the generic inverse-variance method with their corresponding 95% confidence intervals (CIs). To support the primary findings of HR, odds ratios (ORs) calculated via the Mantel-Haenszel method were strictly presented as supplementary sensitivity analyses. For the assessment of adverse events, only ORs and 95% CIs were employed. To ensure all data were in a consistent format for synthesis, conversion methods were applied. HRs and their 95% CIs were log-transformed to obtain the ln (HR) and its corresponding standard error (SE), enabling the use of the generic inverse-variance method. The SE was calculated using the formula: $SE=\frac{\ln\left(upper\;CI\;\right)-\ln\left(lower\;CI\right)}{3.92}$ . Heterogeneity was assessed using the I^2^ statistic, with significant heterogeneity defined as I^2^ > 50%. After initial fixed-effects model analysis revealed a high degree of heterogeneity between studies (I^2^>50%) for subgroup analysis, a meta-analysis was performed using a random-effects model. Publication bias was assessed using funnel plot. However, in accordance with the recommendations of the Cochrane Handbook for Systematic Reviews of Interventions, formal statistical tests for publication bias (e.g., Egger’s test) were not conducted because fewer than 10 studies were included in the meta-analysis. Statistical significance was defined as a two-sided P < 0.05.

### 2.6 Protocol deviations

This systematic review and meta-analysis was conducted in strict accordance with the PRISMA guidelines. As data extraction and methodology evolved, minor deviations from our initial PROSPERO protocol (CRD420251145736) were implemented to optimize scientific rigor. Firstly, progression-free survival (PFS) was omitted to put the focus on the most definitive clinical endpoints (OS and MFS). Moreover, a formal GRADE assessment was additionally incorporated to evaluate the certainty of evidence. Additionally, STATA software was omitted as Review Manager 5.4 was sufficient for all quantitative analyses. Finally, although Egger’s test was more specific, it was not performed because the final number of trials was fewer than 10. These refinements have been transparently updated in our PROSPERO registration.

## 3 Results

### 3.1 Literature search process and results

**FIGURE 1:**
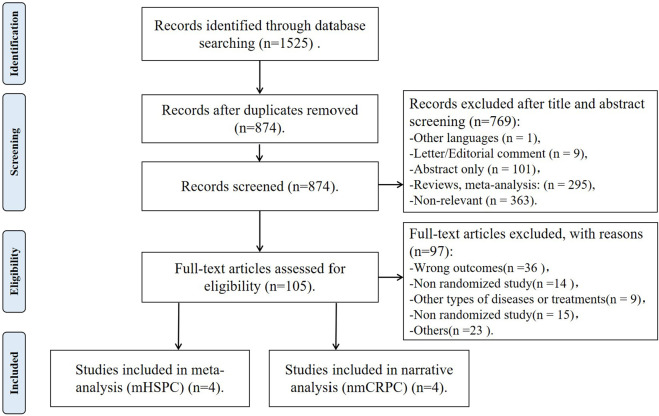
PRISMA flow chart of the current study. The figure shows the flow of study identification and selection. Systematic database searches identified 1,525 records. After removing 651 duplicates, 874 unique records remained for title and abstract screening, excluding 769. The remaining 105 full-text articles were assessed for eligibility. Ultimately, eight articles derived from three trials met all inclusion criteria. Of these, four articles were included in the meta-analysis for the mHSPC cohort, and four articles were included in the narrative analysis for the nmCRPC cohort.

Our initial search identified 1,525 records, and after removing duplicates, 874 remained (Figure 1). A total of 769 articles were excluded after screening the titles and abstracts, and a full-text review was performed for 105 articles. Based on the selection criteria, we identified eight articles from three independent phase III trials (Fizazi et al., 2019; Smith et al., 2022; Saad et al., 2024; Hussain et al., 2023; Shore et al., 2024; Fizazi et al., 2020; Uemura et al., 2021; Shore et al., 2022). There were 3,483 patients involved: 1,509 nmCRPC patients from the ARAMIS trial (Fizazi et al., 2019; Fizazi et al., 2020; Uemura et al., 2021; Shore et al., 2022), 1,974 mHSPC patients from the ARASENS (Smith et al., 2022; Hussain et al., 2023; Shore et al., 2024) and ARANOTE trials (Saad et al., 2024). In the ARASENS trial, patients were randomized to darolutamide plus docetaxel and ADT or placebo plus docetaxel and ADT. In the ARAMIS and ARANOTE trials, patients were randomized to darolutamide plus ADT or placebo plus ADT. For the mHSPC cohort (4 articles from two trials) (Smith et al., 2022; Saad et al., 2024; Hussain et al., 2023; Shore et al., 2024), a meta-analysis was performed. For the nmCRPC cohort (4 articles from one trial) (Fizazi et al., 2019; Fizazi et al., 2020; Uemura et al., 2021; Shore et al., 2022), a narrative synthesis was conducted. To evaluate the efficacy of darolutamide across different stages of prostate cancer, we categorized the trials and their associated articles into two distinct groups based on disease classification: the nmCRPC group and the mHSPC group. The baseline characteristics of the included studies are highlighted in Table 1, and the results of the extracted endpoints are presented in Table 2.

**TABLE 1:** Baseline characteristics of the included studies.

Study/Author	Year	Period	Country	Treatment 1	Treatment 2	Disease status	Median follow-up	Median duration of exposure	Median age	No. Of patients	No. Of patients (T1)	No. Of patients (T2)
ARAMIS Fizazi et al.	2019	2014–2018	North America Asia-pacific Rest of the world	Darolutamide 600 mg + ADT	Placebo + ADT	nmCRPC	17.9 mon	T1:14.8 mon T2:11.0 mon	T1:74 (48–95) T2:74 (50–92)	1,509	955	554
ARAMIS Fizazi et al.	2020	2014–2019	North America Asia-pacific Rest of the world	Darolutamide 600 mg + ADT	Placebo + ADT	nmCRPC	29.0 mon	25.8 mon	T1:74 (48–95) T2:74 (50–92)	1,509	955	554
ARAMIS Uemura et al.	2020	2014–2018	Japan	Darolutamide 600 mg + ADT	Placebo + ADT	nmCRPC	17.9 mon	T1:14.8 mon T2:11.0 mon	T1:77 (56–90) T2:76 (56–87)	95	62	33
ARAMIS Shore et al.	2022	2014–2019	African-american	Darolutamide 600 mg + ADT	Placebo + ADT	nmCRPC	29.0 mon	25.8 mon	T1:72.5 (60–85) T2:72 (55–90)	52	28	24
ARANOTE Fizazi et al.	2024	2021–2024	Asia Latin american Europe and rest of the world	Darolutamide 600 mg + ADT	Placebo + ADT	mHSPC	T1:25.3 mon T2:25.0 mon	T1:24.2 mon T2:17.3 mon	T1:70 (43–93) T2:70 (45–91)	669	446	223
ARASENS Smith et al.	2022	2016–2021	North America Asia-pacific Rest of the world	Darolutamide 600 mg + Docetaxel + ADT	Placebo + Docetaxel + ADT	mHSPC	T1:43.7 mon T2:42.4 mon	T1:41.0 mon T2:16.7 mon	T1:67 (41–89) T2:67 (42–86)	1,305	651	654
ARASENS Hussain et al.	2023	2016–2021	North America Asia-pacific Rest of the world	Darolutamide 600 mg + Docetaxel + ADT	Placebo + Docetaxel + ADT	mHSPC	T1:43.7 mon T2:42.4 mon	T1:41.0 mon T2:16.7 mon	T1:67 (41–89) T2:67 (42–86)	1,302	652	650
ARASENS Shore et al.	2024	2016–2021	North America Asia-pacific Rest of the world	Darolutamide 600 mg + Docetaxel + ADT	Placebo + Docetaxel + ADT	mHSPC	T1:43.7 mon T2:42.4 mon	T1:29.6 mon T2:12.4 mon	T1:63.5 (52–84) T2:63 (47–82)	54	26	28

*ADT*, androgen deprivation therapy; *nmCRPC*, non-metastatic castration-resistant prostate cancer; *mHSPC*, metastatic hormone-sensitive prostate cancer.

**TABLE 2:** Results of the extracted endpoints.

Study/Author	Year	OS/HR	MFS/HR	Any AE	Serious AE	Grade 3 or 4 AE	Grade5 AE
ARAMIS Fizazi et al.	2019	T1:78 T2:58 0.71 (0.50–0.99)	T1:221 T2:216 0.41 (0.34–0.50)	T1:794 T2:426	T1:237 T2:111	T1:236 T2:108	T1:37 T2:18
ARAMIS Fizazi et al.	2020	T1:148 T2:106 0.69 (0.53–0.88)	NA	T1:818 T2:439	T1:249 T2:121	T1:251 T2:120	T1:38 T2:19
ARAMIS Uemura et al.	2020	T1:3 T2:2 0.72 (0.12–4.31)	T1:9 T2:11 0.28 (0.11–0.70)	T1:53 T2:21	T1:20 T2:3	T1:16 T2:4	NA
ARAMIS Shore et al.	2022	T1:1 T2:7 0.09 (0.01–0.72)	T1:1 T2:10 OR:0.055 (0.001–0.452)	T1:23 T2:22	NA	T1:7 T2:2	T1:0 T2:3
ARANOTE Fizazi et al.	2024	T1:103 T2:60 0.81 (0.59 to 1.12)	NA	T1:405 T2:199	T1:105 T2:52	T1:137 T2:67	T1:21 T2:12
ARASENS Smith et al.	2022	T1:229 T2:304 0.68 (0.57–0.80)	NA	T1:649 T2:643	T1:292 T2:275	NA	T1:27 T2:26
ARASENS Hussain et al.	2023	NA	NA	T1:649 T2:643	T1:292 T2:275	NA	T1:27 T2:26
ARASENS Shore et al.	2024	NA	NA	T1:26 T2:27	T1:11 T2:7	T1:16 T2:17	T1:2 T2:0

*OS*, overall survival; *MFS*, metastasis-free survival; *AE*, adverse event; *HR*, hazard ratio; *NA* not available.

### 3.2 The prognosis of darolutamide combination therapy by meta-analysis

#### 3.2.1 mHSPC

**FIGURE 2:**
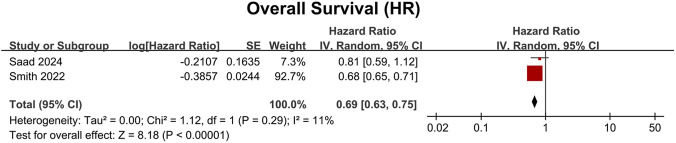
Forest plot of overall survival (OS) by hazard ratio (HR) for darolutamide versus the control group in the mHSPC cohort. The red squares represent the HR for each individual study, with the size of the square proportional to the study’s weight in the meta-analysis. The horizontal lines indicate the 95% confidence intervals (CIs). The black diamond represents the pooled overall HR, calculated using a random-effects model via the generic inverse-variance method. CI confidence interval, HR hazard ratio, IV inverse variance, mHSPC metastatic hormone-sensitive prostate cancer, OS overall survival, SE standard error.

To assess the efficacy of darolutamide in patients with mHSPC, we conducted a meta-analysis from two trials (Smith et al., 2022; Saad et al., 2024). Data of 1974 patients showed that darolutamide plus ADT with or without docetaxel significantly improved OS compared to placebo plus ADT with or without docetaxel (HR: 0.69, 95% CI 0.63–0.75, P < 0.00001) (Figure 2). Importantly, this robust OS benefit was further confirmed by our sensitivity analysis using odds ratio (OR: 0.68, 95% CI 0.53–0.88, P = 0.003) (Supplementary Figure S1).

#### 3.2.2 nmCRPC

Consistent with the mHSPC cohort, a similar efficacy profile was observed among nmCRPC patients. We narratively synthesized the findings from the four articles derived from the ARAMIS trial (Fizazi et al., 2019; Fizazi et al., 2020; Uemura et al., 2021; Shore et al., 2022). A detailed summary of these efficacy endpoints is presented in Table 3; Supplementary Table S3. In the primary analysis of the ARAMIS trial (Fizazi et al., 2019), darolutamide demonstrated a significant improvement in overall survival (HR: 0.71, 95% CI 0.50–0.99, P = 0.045) and metastasis-free survival (HR: 0.41, 95% CI 0.34–0.50, P < 0.001), compared to placebo. Additionally, the extended follow-up analysis confirmed the survival benefit of darolutamide (HR: 0.69, 95% CI 0.54–0.88, P = 0.003) (Fizazi et al., 2020). Further reports from the ARAMIS trial confirmed that this efficacy was consistent across different ethnic and regional subgroups. Specifically, Uemura et al. reported a robust MFS benefit (HR: 0.28, 95% CI 0.11–0.70) alongside a trend favoring darolutamide in OS (HR: 0.72, 95% CI 0.12–4.31) within the Japanese subgroup (Uemura et al., 2021). Similarly, in an analysis of African American patients, Shore et al. observed comparable efficacy, demonstrating prolonged OS (HR: 0.09, 95% CI 0.01–0.72) (Shore et al., 2022). Importantly, sensitivity analyses evaluating these efficacy outcomes via odds ratios (ORs) yielded highly consistent results across all the studies, affirming the robustness of the treatment benefit (Supplementary Table S3).

**TABLE 3:** Summary tables of efficacy outcomes related to narrative synthesis of overall survival (OS) and metastasis-free survival (MFS) by hazard ratios (HR) for nmCRPC patients.

Study (Year)	Population/Subgroup	Median follow-up (months)	OS (HR, 95% CI, P-value)	MFS (HR, 95% CI, P-value)
Fizazi 2019	Overall (primary)	17.9	0.71 (0.50–0.99), P = 0.045	0.41 (0.34–0.50), P < 0.001
Fizazi 2020	Overall (extended)	29	0.69 (0.54–0.88), P = 0.003	NR, P = NR
Uemura 2020	Japanese subgroup	17.9	0.72 (0.12–4.31), P = NR	0.28 (0.11–0.70), P = NR
Shore 2022	Black/African-american	29	0.09 (0.01–0.72), P = NR	NR, P = NR

*nmCRPC*, non-metastatic castration-resistant prostate cancer; *OS*, overall survival; *MFS*, metastasis-free survival; *HR*, hazard ratio; *NR*, not reported.

### 3.3 The overall survival in mHSPC by subgroup meta-analysis based on clinical parameters

To elucidate the efficacy of darolutamide in subgroups, we conducted subgroup meta-analyses of OS among mHSPC patients for baseline total PSA (tPSA) values, Gleason score, age, race, and ECOG PS from two studies (Smith et al., 2022; Saad et al., 2024).

#### 3.3.1 tPSA value

**FIGURE 3:**
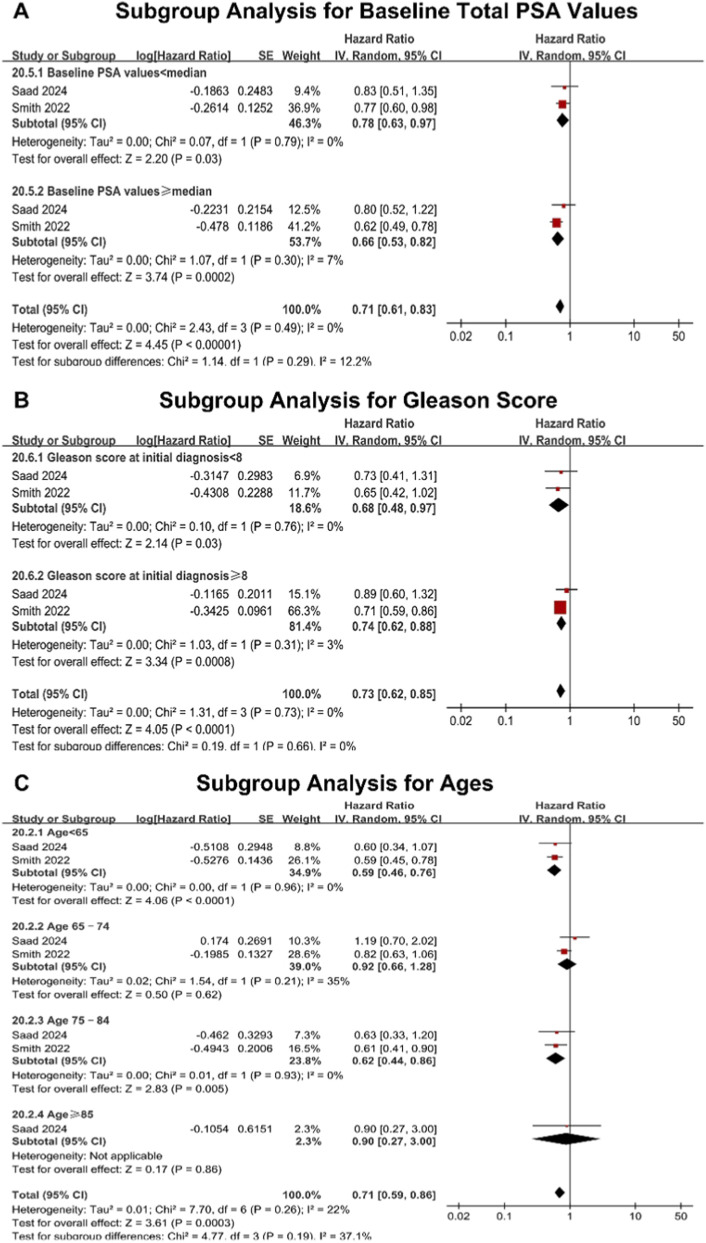
Subgroup analyses of overall survival (OS) by hazard ratio (HR) for darolutamide versus the control group in the mHSPC cohort. **(A)** The subgroup analysis of treatment effect for baseline total prostate-specific antigen (PSA) values. The subgroups are defined as PSA levels lower than the median or higher than the median of the overall study population. **(B)** The subgroup analysis of treatment effect for Gleason score. The subgroups are defined as Gleason score lower than eight or higher than 8. **(C)** The subgroup analysis of treatment effect for ages. The subgroups are defined as age <65, age ≥65 and <75, age ≥75 and <85, age ≥85. The red squares represent the HR for each individual study, with the size of the square proportional to the study’s weight in the meta-analysis. The horizontal lines indicate the 95% confidence intervals (CIs). The black diamonds represent the pooled HRs for each subgroup and the overall cohort, calculated using a random-effects model via the generic inverse-variance method. CI confidence interval, HR hazard ratio, IV inverse variance; mHSPC metastatic hormone-sensitive prostate cancer, OS overall survival, PSA prostate-specific antigen, SE standard error.

Firstly, for baseline tPSA values, darolutamide combined with docetaxel and ADT demonstrated satisfactory efficacy in decreasing the risk of death in patients both with tPSA values < median (HR: 0.78, 95% CI 0.63–0.97, P = 0.03; OR: 0.75, 95% CI 0.57–0.99, P = 0.04) and ≥ median (HR: 0.66, 95% CI 0.53–0.82, P = 0.0002; OR: 0.62, 95% CI 0.41–0.96, P = 0.03) (Figure 3A; Supplementary Figure S3A).

#### 3.3.2 Gleason score

We subsequently analyzed the OS effect between groups with the Gleason score. The OS benefit from adding darolutamide to docetaxel plus ADT treatment was confirmed among patients with a Gleason score ≥ 8 (HR: 0.74, 95% CI 0.62–0.88, P = 0.0008; OR: 0.71, 95% CI 0.53–0.96, P = 0.03) and < 8 (HR: 0.68, 95% CI 0.48–0.97, P = 0.03; OR: 0.66, 95% CI 0.43–1.01, P = 0.06). This marginal loss of significance in the OR analysis is likely attributable to the combination of a smaller sample size and the inherently lower mortality rate in this subgroup with lower risk, warranting larger cohorts for definitive validation (Figure 3B; Supplementary Figure S3B).

#### 3.3.3 Age

Additionally, for ages, the OS improvement was more evident among patients aged under 65 who received darolutamide plus ADT with or without docetaxel (HR: 0.59, 95% CI 0.46–0.76, P < 0.0001; OR: 0.51, 95% CI 0.37–0.70, P < 0.0001) and 75 to 84 (HR: 0.62, 95% CI 0.44–0.86, P = 0.005; OR: 0.56, 95% CI 0.36–0.87, P = 0.01). However, there was no statistical difference in patients aged between 65 and 74 (HR: 0.92, 95% CI 0.66–1.28, P = 0.62; OR: 0.92, 95% CI 0.62–1.35, P = 0.67) or over 85 (HR: 0.90, 95% CI 0.27–3.00, P = 0.86; OR: 1.08, 95% CI 0.26–4.44, P = 0.92). The lack of significance in the 65-74 group may stem from internal heterogeneity, while the wide confidence intervals in the ≥ 85 group reflect the severely limited sample size (Figure 3C; Supplementary Figure S3C).

#### 3.3.4 Race and ECOG performance status

When stratified by race, only the White subgroup reached statistical significance for OS benefit (HR: 0.66, 95% CI 0.54–0.81, P < 0.0001; OR: 0.62, 95% CI 0.48–0.81, P = 0.0004), a result largely driven by the predominant enrollment of White patients and inadequate sample sizes in other racial subgroups. Regarding the Eastern Cooperative Oncology Group (ECOG) performance status, the OS benefit of darolutamide was consistent across both ECOG PS = 0 (HR: 0.77, 95% CI 0.63–0.93, P = 0.008; OR: 0.73, 95% CI 0.57–0.93, P = 0.01) and ECOG PS ≥ 1 (HR: 0.68, 95% CI 0.47–0.97, P = 0.03; OR: 0.61, 95% CI 0.38–0.99, P = 0.05) (Supplementary Figure S2; Supplementary Figures S3D–E).

### 3.4 The safety assessment of darolutamide combination therapy by meta-analysis

#### 3.4.1 mHSPC

**FIGURE 4:**
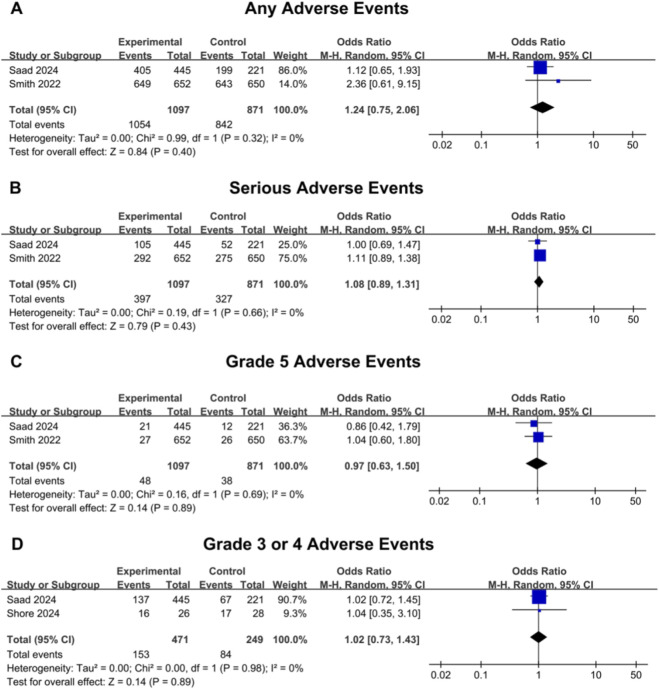
Forest plots of outcomes related to adverse events of the darolutamide group versus the control group for mHSPC patients. **(A)** The plot for any adverse events. **(B)** The plot for serious adverse events. **(C)** The results for grade 5 adverse events. **(D)** The results for grade 3 or four adverse events. The blue squares represent the OR for each individual study, with the size of the square proportional to the study’s weight in the meta-analysis. The horizontal lines indicate the 95% confidence intervals (CIs). The black diamonds represent the pooled overall ORs, calculated using a random-effects model via the Mantel-Haenszel method. CI confidence interval, mHSPC metastatic hormone-sensitive prostate cancer, M-H Mantel-Haenszel, OR odds ratio.

For mHSPC events, no significant differences were observed between the darolutamide and placebo groups concerning any adverse events (OR: 1.24, 95% CI 0.75–2.06, P = 0.40) (Figure 4A) and serious adverse events (OR: 1.08, 95% CI 0.89–1.31, P = 0.43) (Figure 4B). Similarly, when narrowing down to various grades of adverse events, the analysis did not reveal notable differences between the placebo and darolutamide groups in terms of both grade 5 adverse events (OR: 0.97, 95% CI 0.63–1.50, P = 0.89) (Figure 4C) and three or four adverse events (OR: 1.02, 95% CI 0.73–1.43, P = 0.89) (Figure 4D). Furthermore, regarding the incidence of specific adverse events, no differences were observed between these two groups, including hypertension (OR: 1.30, 95% CI 0.89–1.92, P = 0.18), anemia (OR: 1.16, 95% CI 0.94–1.44, P = 0.17), constipation (OR: 1.19, 95% CI 0.93–1.52, P = 0.16), back pain (OR: 0.98, 95% CI 0.77–1.25, P = 0.87), pain in extremity (OR: 1.20, 95% CI 0.91–1.59, P = 0.20), arthralgia (OR: 1.04, 95% CI 0.84–1.30, P = 0.71), fatigue (OR: 0.92, 95% CI 0.65–1.29, P = 0.62), and hot flush (OR: 1.06, 95% CI 0.82–1.37, P = 0.64) (Supplementary Figure S4).

#### 3.4.2 nmCRPC

Similarly, safety outcomes for the nmCRPC cohort derived from the ARAMIS trial were evaluated via narrative synthesis. An overview of the overall adverse events is presented in Table 4, while the data regarding specific adverse events are detailed in Supplementary Table S4. Based on the primary analysis (Fizazi et al., 2019), the addition of darolutamide slightly increased the overall odds of any adverse events (OR: 1.49, 95% CI: 1.15–1.93) and grade 3 or four adverse events (OR: 1.36, 95% CI: 1.05–1.75). Reassuringly, the extended follow-up analysis demonstrated that this safety profile remained stable over longer exposure (Fizazi et al., 2020). Importantly, the mature extended data confirmed that darolutamide did not significantly increase the risk of serious adverse events (Extended OR: 1.26, 95% CI: 0.99–1.62) or grade 5 adverse events (Extended OR: 1.17, 95% CI: 0.67–2.05). Regarding specific adverse events, the darolutamide combination showed incidences comparable to placebo for the majority of events, including hypertension, bone fracture, falls, anemia, urinary tract infection, weight decrease, diarrhea, constipation, back pain, arthralgia, nausea, hot flush, and cardiovascular events such as heart failure. Interestingly, darolutamide was associated with an elevated risk of rash (Extended OR: 2.97, 95% CI: 1.23–7.17), fatigue (Extended OR: 1.68, 95% CI: 1.18–2.40), and pain in extremity (Primary OR: 1.82, 95% CI: 1.06–3.14) (Supplementary Table S4). Subsequent subgroup analyses in Japanese and African American populations revealed generally consistent safety trends. Overall, darolutamide combined with ADT demonstrated a highly tolerable safety profile without increasing the risk of fatal adverse events in nmCRPC patients.

**TABLE 4:** Summary tables of safety outcomes related to general categories of adverse events (AEs) for nmCRPC patients.

Adverse events categories (OR, 95% CI)	Fizazi 2019 (primary, 17.9 m)	Fizazi 2020 (extended, 29.0 m)	Uemura 2020 (Japanese)	Shore 2022 (Black/African-American)
Any AE	1.49 (1.15–1.93)	1.58 (1.20–2.07)	3.37 (1.24–9.16)	0.42 (0.07–2.38)
Serious AE	1.32 (1.02–1.70)	1.26 (0.99–1.62)	4.76 (1.30–17.49)	NR
Grade 3 or 4 A E	1.36 (1.05–1.75)	1.29 (1.01–1.66)	NR	3.67 (0.68–19.70)
Grade 5 A E	1.20 (0.68–2.13)	1.17 (0.67–2.05)	NR	0.11 (0.01–2.20)

*nmCRPC*, non-metastatic castration-resistant prostate cancer; *AE*, adverse event; *OR*, odds ratio; *NR*, not reported.

### 3.5 Heterogeneity and risk of bias analysis

**FIGURE 5:**
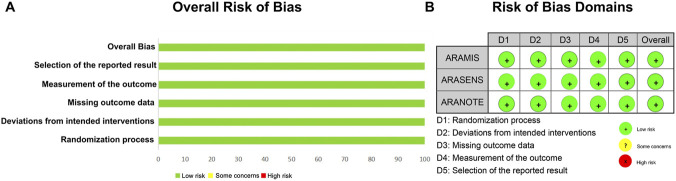
Risk of bias assessment for the included phase III trials using the revised Cochrane risk-of-bias tool (RoB 2). **(A)** The plot for the overall risk of bias. **(B)** The plot for the risk of bias domains.

The risk of bias for the included trials was assessed using the revised Cochrane risk-of-bias tool for randomized trials (RoB 2) (Figure 5). Funnel plots for the reported outcomes appeared symmetrical, indicating no significant publication bias (Supplementary Figures S5, S6). Most prognostic outcomes demonstrated low heterogeneity across studies (I^2^ = 11%) (Figure 2). However, moderate heterogeneity was observed in the supplementary overall survival using OR in mHSPC patients (I^2^ = 32%) (Supplementary Figure S1). To investigate the potential sources of heterogeneity for OS in mHSPC patients, subgroup analyses were conducted based on baseline tPSA, Gleason score, age, race, and ECOG PS. Interestingly, heterogeneity persisted in the age subgroups (I^2^ > 30%), indicating a potential interaction effect (Figure 3C). This variance in efficacy across age subgroups may be due to the different tumor biology and physical fitness, which are explained in the discussion. Regarding safety assessments, heterogeneity was generally low. Moderate heterogeneity was also noted in specific adverse events, including hypertension for mHSPC patients (I^2^ = 31%) (Supplementary Figure S4G).

### 3.6 GRADE rating

The GRADE rating for important categorical and continuous variables in advanced prostate cancer patients classified the evidence as follows. Because all included studies were randomized controlled trials, the baseline certainty of evidence was initially rated as high. High-certainty evidence was firmly maintained for Overall Survival and Metastasis-free Survival (assessed with HR and OR) in both the nmCRPC and mHSPC cohorts, as well as for Any adverse events, Serious adverse events, and Grade 3 or four adverse events. However, the evidence for Grade 5 adverse events in both the nmCRPC and mHSPC cohorts was downgraded to moderate certainty. Based on the GRADE criteria, this specific downgrade was primarily due to imprecision originating from the low number of patients experiencing these adverse events, which leads to wider confidence intervals. Detailed GRADE ratings are provided in Supplementary Table S2.

## 4 Discussion

In this meta-analysis, we evaluated the clinical effectiveness and safety of systemic treatments for patients with nmCRPC and mHSPC. Our findings indicate that, compared with placebo, adding darolutamide significantly reduced the risk of death and prolonged MFS in patients with both nmCRPC and mHSPC. As for subgroups, our conclusions are equally applicable. According to the results of safety outcomes, for nmCRPC events, the incidences of serious adverse events, such as heart failure, bone fracture, fall, and hypertension, showed no statistical difference between darolutamide and placebo. And there is no significant difference between darolutamide and placebo for mHSPC events among any grades’ adverse events or various specific adverse effects. Collectively, this meta-analysis suggests that darolutamide combination therapy confers a survival benefit with a favorable safety profile in patients with advanced prostate cancer.

The combination of darolutamide and ADT offers clinical benefits for patients with nmCRPC. However, there remains insufficient evidence to definitively establish its superiority over other androgen receptor pathway inhibitors (ARPIs). A network meta-analysis conducted by Wenzel et al. indicated that darolutamide was associated with a higher OS compared with apalutamide and enzalutamide in the nmCRPC population. Notably, in the subgroup of patients with a prostate-specific antigen doubling time (PSA-DT) of lower than 6 months, enzalutamide demonstrated the greatest efficacy (Wenzel et al., 2022). In contrast, a study by Halabi et al. reported no significant differences in metastasis-free survival (MFS) among darolutamide, apalutamide, and enzalutamide in nmCRPC patients (Halabi et al., 2021). Wang et al. further suggested through a network meta-analysis that abiraterone acetate may provide MFS benefits comparable with those of darolutamide in this patient population (Wang et al., 2022). Additionally, a meta-analysis by Roumiguié et al. confirmed that darolutamide, enzalutamide, and apalutamide significantly prolong both MFS and OS, along with substantially improving PSA response rates (Roumiguie et al., 2021). Nevertheless, some studies have raised questions about the relative efficacy of darolutamide. For instance, a network meta-analysis by Chen et al. found that while enzalutamide, apalutamide, and darolutamide all improve MFS, enzalutamide and apalutamide exhibit superior effectiveness compared to darolutamide (Chen et al., 2023). Our results align with the consensus that adding darolutamide to ADT significantly improves OS and MFS in nmCRPC, though head-to-head comparisons are still needed to clarify relative efficacy (Table 3; Supplementary Table S3).

In the mHSPC population, ADT and ARPI combination therapy demonstrates superior efficacy compared with ADT alone. However, it remains uncertain whether ADT plus ARPI with docetaxel triple therapy is more effective than ADT plus ARPI dual therapy. And there is currently insufficient evidence to determine the relative efficacy of darolutamide compared with other ARPIs. Multiple meta-analyses have demonstrated that, when added to the dual therapy regimen of docetaxel plus ADT, the inclusion of ARPIs, such as darolutamide, abiraterone acetate, apalutamide, and enzalutamide, confers additional OS benefits (Yanagisawa et al., 2022; Matsukawa et al., 2024; Dou et al., 2023). Yanagisawa et al. conducted a meta-analysis and reported that triple therapy incorporating darolutamide is associated with improved OS outcomes compared to dual therapy with ADT plus ARPI (Yanagisawa et al., 2022). Similarly, a network meta-analysis evaluating the surface under the cumulative ranking curve (SUCRA) indicated that darolutamide-based triple therapy has the highest SUCRA value for OS, suggesting superior ranking among both triple and dual ARPI-containing regimens (Wang S. et al., 2024). In contrast, the meta-analysis by Irbaz et al. found no statistically significant OS advantage for darolutamide-containing triple therapy over ADT plus ARPI dual therapy (Riaz et al., 2023). Furthermore, a benefit-risk assessment by Dominik et al. indicated that the probability of achieving a net clinical benefit with darolutamide-based triple therapy is relatively low (<40%) (Menges et al., 2022). In this meta-analysis, we found that the triple therapy of darolutamide plus ADT with or without docetaxel significantly prolonged OS in patients with mHSPC (Figure 2).

Among patients with mHSPC across various disease strata, darolutamide demonstrates superior efficacy compared to other ARPIs. Multiple meta-analyses have shown that in patients with high-volume mHSPC, the addition of darolutamide to triplet regimen is associated with improved OS (Matsukawa et al., 2024; Wang T. et al., 2024; Hoeh et al., 2025; Hoeh et al., 2023). However, there was no such stratification in the ARASENS trial, because most patients who were enrolled had metastatic disease, with bone metastases, visceral metastases, or both, at the time of the initial diagnosis. Consequently, the specific efficacy of darolutamide based on tumor volume could not be differentiated in our analysis, underscoring the need for future trials to address this gap. Notably, network meta-analyses conducted by Hoeh et al. (2025), Hoeh et al. (2023) and Mandel et al. (2023) indicate that darolutamide-based triplet therapy yields better OS outcomes than dual therapy with ADT plus ARPI alone. Furthermore, the network meta-analysis by Dou et al. demonstrated that darolutamide-containing triplet therapy significantly enhances OS in patients with a Gleason score of ≥8, outperforming regimens involving apalutamide, enzalutamide, and abiraterone (Dou et al., 2023). This result was also verified in our subgroup study for patients with a Gleason score of ≥8 (Figure 3B). As for the ages, although it is clinically expected for prostate cancer to be seen less frequently in patients under 65 years, it tends to be more aggressive. However, younger patients under 65 years usually present with better physical fitness compared to older patients. Due to the better tolerance of systemic treatments, they are more likely to fully benefit from the androgen receptor inhibition of darolutamide. This effectively suppresses the aggressive tumor biology, explaining the substantial survival gains seen in this younger cohort. This may explain why the OS improvement was more evident among patients aged under 65 who received darolutamide plus ADT with or without docetaxel in our study (Figure 3C). Additionally, Zhou et al. suggest that elderly patients (>70 years) with mHSPC also derive a significant OS benefit from darolutamide-based triplet therapy (Zhou et al., 2025). In our study, the patients aged from 75 to 84 derive a significant OS benefit, while the subgroup older than 85 years showed no significant differences (Figure 3C). This lack of significance may be attributed to the small sample size in this specific age subgroup rather than a lack of efficacy, though further verification is required.

Darolutamide has consistently demonstrated a favorable safety profile across several clinical studies (Turco et al., 2024). This dual advantage of efficacy and safety is attributed to its unique molecular structure. Unlike conventional androgen receptor antagonists, which can paradoxically convert into agonists in the presence of resistance mutations like AR (F876L), darolutamide retains full antagonistic activity against these mutations (Bleve et al., 2025; Moilanen et al., 2015). Furthermore, darolutamide exhibits minimal interference with the CYP enzyme system, reducing the risk of drug-drug interactions. Importantly, it possesses an extremely low brain-to-plasma concentration ratio, leading to a significantly lower blood-brain barrier penetration compared to other second-generation AR antagonists (Wenzel et al., 2022). Although direct comparative data among androgen receptor pathway inhibitors (ARPIs) remain limited, multiple systematic reviews and network meta-analyses suggest that darolutamide exhibits superior tolerability. A systematic review and network meta-analysis by Mori et al. in the nmCRPC population showed that, compared with placebo, darolutamide was associated with a similar incidence of fatal adverse events and treatment discontinuations due to toxicity, whereas apalutamide and enzalutamide were linked to increased risks for both outcomes (Mori et al., 2020). Consistent with these indirect comparisons, our narrative synthesis of the nmCRPC patients confirmed that while the addition of darolutamide was associated with slightly higher incidences of specific adverse events like fatigue, it did not significantly increase the risk of serious adverse events or grade 5 adverse events (Table 4). In a network meta-analysis by Wenzel et al., darolutamide was found to have a lower incidence of grade 3 or higher adverse events compared to apalutamide and enzalutamide in the nmCRPC setting (Wenzel et al., 2022). The aforementioned low blood-brain barrier penetration directly translates to a lower risk of central nervous system-related events, such as dizziness and mental disorders, for patients receiving darolutamide (Borgmann et al., 2018). Accordingly, using a matched-adjusted indirect comparison approach, Halabi et al. reported that, in nmCRPC patients, the darolutamide group had statistically significantly lower incidences of falls, fractures, and rashes than the apalutamide group, as well as significantly lower rates of falls, dizziness, mental disorders, fatigue, and severe fatigue compared to the enzalutamide group (Halabi et al., 2021). Wang et al. conducted a network meta-analysis indicating that, relative to placebo, darolutamide conferred a lower risk of serious adverse events than apalutamide, enzalutamide, and abiraterone acetate in the nmCRPC population (Wang et al., 2022). In the mHSPC population, a meta-analysis of triplet therapy by Yanagisawa et al. demonstrated that while the addition of abiraterone to ADT plus docetaxel increased the incidence of grade higher than three adverse events, the addition of darolutamide to the same backbone did not result in a similar increase (Yanagisawa et al., 2022). Our outcomes for mHSPC also show that adding darolutamide did not increase the incidence of adverse events statistically (Figure 4).

The following limitations of the current study need to be discussed. Firstly, regarding the nmCRPC cohort, the available evidence was derived from multiple overlapping publications originating from a single randomized controlled trial (ARAMIS). To strictly avoid unit-of-analysis errors, we were restricted to performing a narrative synthesis. Next, in the mHSPC analysis, we pooled data from composite studies where patients received different treatments, specifically doublet and triplet therapy regimens. Although our statistical heterogeneity was controlled, combining these different treatment combinations inevitably introduces methodological heterogeneity. Moreover, during the subgroup analyses for the mHSPC cohort, certain patient subgroups (such as those aged >85 years) suffered from extremely limited sample sizes. Consequently, the statistical power was insufficient to draw valid conclusions regarding the efficacy of darolutamide in these subgroups. Meanwhile, the populations enrolled in the clinical trials were predominantly from regions such as North America, Europe, and Japan. This underrepresentation of other ethnic groups introduces a potential racial bias, limiting the global generalizability of our findings. Future research should extend to more diverse countries to ensure broader inclusivity. In addition, our analysis exclusively evaluated darolutamide against placebo within standard therapy. Because darolutamide has only recently entered broader clinical practice compared to earlier antiandrogens, there is a lack of head-to-head comparative studies between darolutamide and other next-generation androgen receptor inhibitors. Finally, owing to the relatively small number of eligible trials (n < 10), we were unable to perform formal statistical tests (Egger’s test) to rule out publication bias, relying on subjective funnel plots. Additionally, restricting our literature search exclusively to English-language databases introduces a potential language bias, risking the omission of non-English research.

## 5 Conclusion

In conclusion, our systematic review and meta-analysis have shown that the addition of darolutamide in ADT with/without docetaxel significantly prolongs survival compared with placebo in both patients with mHSPC and nmCRPC. Simultaneously, the consistent efficacy across subgroups highlights the promising potential of darolutamide for patients with different clinical and biological characteristics. Due to its efficacy and safety, darolutamide can be considered as an alternative option.

## Data Availability

The original contributions presented in the study are included in the article/Supplementary Material, further inquiries can be directed to the corresponding authors.
