# Supplementary material for: The efficacy and safety of darolutamide combination therapy in advanced prostate cancer: a systematic review and meta-analysis of randomized controlled trials
Source: Front Pharmacol. 2026 May 18;17:1818807. doi: 10.3389/fphar.2026.1818807 (PMC13223009; doi:10.3389/fphar.2026.1818807)
Supplement: Supplementary file 1 [file Table1.pdf]

Supplementary Table S1. The search strategies of databases.

Databases	Search strategies
Pubmed	((Neoplasms, Prostatic) OR (Neoplasm, Prostatic) OR (Prostatic Neoplasm) OR (Prostate Neoplasms) OR (Neoplasms, Prostate) OR (Neoplasm, Prostate) OR (Prostate Neoplasm) OR (Prostate Cancer) OR (Cancer, Prostate) OR (Cancers, Prostate) OR (Prostate Cancers) OR (Cancer of Prostate) OR (Cancer of the Prostate) OR (Prostatic Cancer) OR (Cancer, Prostatic) OR (Cancers, Prostatic) OR (Prostatic Cancers) OR ("Prostatic Neoplasms"[Mesh]))<br>AND ((darolutamide) OR (ORM-16555)OR (ODM-201) OR (ORM-16497)OR (Nubeqa))<br>AND ((randomized controlled trial[pt] OR controlled clinical trial[pt] OR clinical trials as topic[mesh:noexp] OR trial[ti] OR random*[tiab] OR placebo*[tiab]))
The Cochrane Library	#1 MeSH descriptor: [Prostatic Neoplasms] explode all trees<br>#2 (Neoplasms, Prostatic):ti,ab,kw (Word variations have been searched)<br>#3 (Neoplasm, Prostatic):ti,ab,kw (Word variations have been searched)<br>#4 (Prostatic Neoplasm):ti,ab,kw (Word variations have been searched)<br>#5 (Prostate Neoplasms):ti,ab,kw (Word variations have been searched)<br>#6 (Neoplasms, Prostate):ti,ab,kw (Word variations have been searched)<br>#7 (Neoplasm, Prostate):ti,ab,kw (Word variations have been searched)<br>#8 (Prostate Neoplasm):ti,ab,kw (Word variations have been searched)<br>#9 (Prostate Cancer):ti,ab,kw (Word variations have been searched)<br>#10 (Cancer, Prostate):ti,ab,kw (Word variations have been searched)<br>#11 (Cancers, Prostate):ti,ab,kw (Word variations have been searched)<br>#12 (Prostate Cancers):ti,ab,kw (Word variations have been searched)<br>#13 (Cancer of Prostate):ti,ab,kw (Word variations have been searched)<br>#14 (Cancer of the Prostate):ti,ab,kw (Word variations have been searched)<br>#15 (Prostatic Cancer):ti,ab,kw (Word variations have been searched)<br>#16 (Cancer, Prostatic):ti,ab,kw (Word variations have been searched)<br>#17 (Cancers, Prostatic):ti,ab,kw (Word variations have been searched)<br>#18 (Prostatic Cancers):ti,ab,kw (Word variations have been searched)<br>#19 #1 or #2 or #3 or #4 or #5 or #6 or #7 or #8 or #9 or #10 or #11 or #12 or #13 or #14 or #15 or #16 or #17 or #18<br>#20 (darolutamide):ti,ab,kw (Word variations have been searched)<br>#21 (ORM-16555):ti,ab,kw (Word variations have been searched)<br>#22 (ODM-201):ti,ab,kw (Word variations have been searched)<br>#23 (ORM-16497):ti,ab,kw (Word variations have been searched)<br>#24 (Nubeqa):ti,ab,kw (Word variations have been searched)

	#25 #20 OR #21 OR #22 OR #23 OR #24<br>#26 #25 AND #19
Web of Science	'prostate cancer' OR 'prostate cancer' OR 'prostate neoplasm' OR 'neoplasm, prostate' OR 'neoplasms, prostate' OR 'prostate neoplasms' OR 'prostatic neoplasm' OR 'neoplasm, prostatic' OR 'neoplasms, prostatic' OR 'prostate cancer' OR 'cancer, prostates' OR 'cancers, prostate' OR 'prostate cancers'OR 'cancer of prostate' OR 'cancer of the prostate' OR 'prostatic cancer' OR 'cancer, prostatic' OR 'cancers, prostatic' OR 'prostatic cancers'OR 'prostatic neoplasms' (All Fields) and 'darolutamide' OR 'darolutamide' OR 'darolutamide' OR 'orm-16555' OR 'odm-201' OR 'orm-16497' (All Fields) and 'randomized controlled trial' OR 'randomized controlled trial' OR 'randomized controlled trial' OR 'controlled clinical trial' OR 'clinical trials as topic' OR 'trial' OR 'random' OR 'placebo'
Embase	('prostate cancer'/exp OR 'prostate cancer' OR 'prostate neoplasm':ab,ti OR 'neoplasm, prostate':ab,ti OR 'neoplasms, prostate':ab,ti OR 'prostate neoplasms':ab,ti OR 'prostatic neoplasm':ab,ti OR 'neoplasm, prostatic':ab,ti OR 'neoplasms, prostatic':ab,ti OR 'prostate cancer':ab,ti OR 'cancer, prostater':ab,ti OR 'cancers, prostate':ab,ti OR 'prostate cancers':ab,ti OR 'cancer of prostate':ab,ti OR 'cancer of the prostate':ab,ti OR 'prostatic cancer':ab,ti OR 'cancer, prostatic':ab,ti OR 'cancers, prostatic':ab,ti OR 'prostatic cancers':ab,ti OR 'prostatic neoplasms':ab,ti) AND ('darolutamide'/exp OR 'darolutamide' OR 'darolutamide':ab,ti OR 'orm-16555':ab,ti OR 'odm-201':ab,ti OR 'orm-16497':ab,ti) AND ('randomized controlled trial'/exp OR 'randomized controlled trial' OR 'randomized controlled trial':ab,ti OR 'controlled clinical trial':ab,ti OR 'clinical trials as topic':ab,ti OR 'trial':ti OR 'random':ab,ti OR 'placebo':ab,ti)
clinicaltrials.gov	AREA[ConditionSearch](Prostatic Neoplasm OR Prostate Neoplasms OR Prostate Neoplasm OR Prostate Cancer OR Prostate Cancers OR Cancer of Prostate OR Cancer of the Prostate OR Prostatic Cancer OR Prostatic Cancers OR Prostatic Neoplasms) AND AREA[BasicSearch](darolutamide OR ORM-16555 OR ODM-201 OR ORM-16497)

Supplementary Table S2. GRADE rating of each outcome.

No. of studies	Outcomes	Metrics	Estimate	95%CI	I^2; P value	Risk of bias	Inconsistency	Indirectness	Imprecision	Publication bias	Plausible confounding	Magnitude of effect	Dose-response gradient	GRADE
1	Overall Survival (nmCRPC)	OR	0.78	0.59, 1.02	NA; P=0.29	Not serious	Not serious	Not serious	Not serious	Undetected	No	No	No	High
1	Overall Survival (nmCRPC)	HR	0.69	0.54, 0.88	NA; P=0.004	Not serious	Not serious	Not serious	Not serious	Undetected	No	No	No	High
1	Metastasis-free Survival (nmCRPC)	OR	0.47	0.38, 0.59	NA; P<0.001	Not serious	Not serious	Not serious	Not serious	Undetected	No	No	No	High
1	Metastasis-free Survival (nmCRPC)	HR	0.41	0.34, 0.50	NA; P<0.001	Not serious	Not serious	Not serious	Not serious	Undetected	No	No	No	High
2	Overall Survival (mHSPC)	OR	0.68	0.53, 0.88	32%; P=0.003	Not serious	Not serious	Not serious	Not serious	Undetected	No	No	No	High
2	Overall Survival (mHSPC)	HR	0.69	0.63, 0.75	11%; P<0.001	Not serious	Not serious	Not serious	Not serious	Undetected	No	No	No	High
1	Any AE (nmCRPC)	OR	1.58	1.20, 2.07	NA; P=0.001	Not serious	Not serious	Not serious	Not serious	Undetected	No	No	No	High
1	Serious AE (nmCRPC)	OR	1.26	0.99, 1.62	NA; P＝0.06	Not serious	Not serious	Not serious	Not serious	Undetected	No	No	No	High
1	Grade5 AE (nmCRPC)	OR	1.17	0.67, 2.05	NA; P＝0.59	Not serious	Not serious	Not serious	serious	Undetected	No	No	No	Moderate
1	Grade 3 or 4 AE (nmCRPC)	OR	1.17	0.67, 2.05	NA; P＝0.04	Not serious	Not serious	Not serious	Not serious	Undetected	No	No	No	High

2	Any AE (mHSPC)	OR	1.24	0.75, 2.06	0%; P＝0.40	Not serious	Not serious	Not serious	Not serious	Undetected	No	No	No	High
2	Serious AE (mHSPC)	OR	1.08	0.89, 1.31	0%; P＝0.43	Not serious	Not serious	Not serious	Not serious	Undetected	No	No	No	High
2	Grade5 AE (mHSPC)	OR	0.97	0.63, 1.50	0%; P＝0.89	Not serious	Not serious	Not serious	serious	Undetected	No	No	No	Moderate
2	Grade 3 or 4 AE (mHSPC)	OR	1.02	0.73, 1.43	0%; P＝0.98	Not serious	Not serious	Not serious	Not serious	Undetected	No	No	No	High

Abbreviations: CI: confidence interval; HR: hazard ratio; OR: odds ratio; NA: Not applicable

Supplementary Table S3. Summary table of supportive outcomes related to sensitivity analysis of overall survival (OS) and metastasis-free survival (MFS) by odds ratios for nmCRPC patients.

Study (Year)	Population / Subgroup	OS (OR, 95% CI)	MFS (OR, 95% CI)
Fizazi 2019	Overall (Primary)	0.76 (0.53–1.09)	0.47 (0.38–0.59)
Fizazi 2020	Overall (Extended)	0.78 (0.59–1.02)	NR
Uemura 2020	Japanese subgroup	0.79 (0.13–4.97)	0.34 (0.12–0.93)
Shore 2022	Black/African-American	0.09 (0.01–0.80)	0.05 (0.01–0.45)

Abbreviations: nmCRPC: non-metastatic castration-resistant prostate cancer; OS: overall survival; MFS: metastasis-free survival; NR: not reported; OR: odds ratio.

Supplementary Table S4. Summary table of safety outcomes related to the incidence of specific adverse events (AEs) for nmCRPC patients.

**Specific Adverse Events (OR, 95% CI, P-value)**	**Fizazi 2019 (Primary, 17.9m)**	**Fizazi 2020 (Extended, 29.0m)**	**Uemura 2020 (Japanese)**	**Shore 2022 (Black/African-American)**
Fatigue	1.44 (1.01–2.06)	1.68 (1.18–2.40)	1.07 (0.09–12.22)	1.83 (0.31–11.02)
Hypertension	1.28 (0.81–2.01)	1.21 (0.80–1.83)	1.07 (0.09–12.22)	2.67 (0.10–68.70)
Bone fracture	NR	1.54 (0.91–2.61)	4.07 (0.48–34.62)	0.85 (0.05–14.39)
Rash	3.32 (1.27–8.65)	2.97 (1.23–7.17)	2.21 (0.24–20.59)	2.67 (0.10–8.65)
Pain in extremity	1.82 (1.06–3.14)	NR	1.07 (0.09–12.22)	NR
Heart failure	NR	2.11 (0.78–5.72)	NR	2.67 (0.10–68.70)
Falling, including accident	0.89 (0.54–1.47)	1.08 (0.67–1.75)	4.74 (0.57–39.67)	2.76 (0.27–28.75)
Anemia	1.24 (0.76–2.03)	NR	3.49 (0.20–78.63)	NR
Urinary tract infection	0.97 (0.60–1.57)	NR	0.52 (0.03–8.67)	NR
Weight decrease	1.67 (0.86–3.25)	1.69 (0.91–3.13)	3.94 (0.20–78.63)	2.67 (0.10–68.70)
Diarrhea	1.25 (0.81–1.95)	NR	0.37 (0.08–1.76)	0.86 (0.29–2.61)
Constipation	1.03 (0.66–1.58)	NR	4.47 (0.57–39.67)	NR
Back pain	0.97 (0.67–1.40)	NR	2.81 (0.31–25.09)	NR

**Specific Adverse Events (OR, 95% CI, P-value)**	**Fizazi 2019 (Primary, 17.9m)**	**Fizazi 2020 (Extended, 29.0m)**	**Uemura 2020 (Japanese)**	**Shore 2022 (Black/African-American)**
Arthralgia	0.87 (0.60–1.25)	NR	0.25 (0.02–2.91)	NR
Nausea	0.86 (0.55–1.37)	NR	5.15 (0.27–98.71)	NR
Hot flush	1.28 (0.77–2.12)	1.34 (0.83–2.18)	1.63 (0.06–41.23)	4.62 (0.21–101.15)

Abbreviations: nmCRPC: non-metastatic castration-resistant prostate cancer; AE: adverse event; NR: not reported; OR: odds ratio.
