# Supplementary material for: The efficacy and safety of darolutamide combination therapy in advanced prostate cancer: a systematic review and meta-analysis of randomized controlled trials
Source: Front Pharmacol. 2026 May 18;17:1818807. doi: 10.3389/fphar.2026.1818807 (PMC13223009; doi:10.3389/fphar.2026.1818807)
Supplement: Supplementary file 2 [file Image1.pdf]

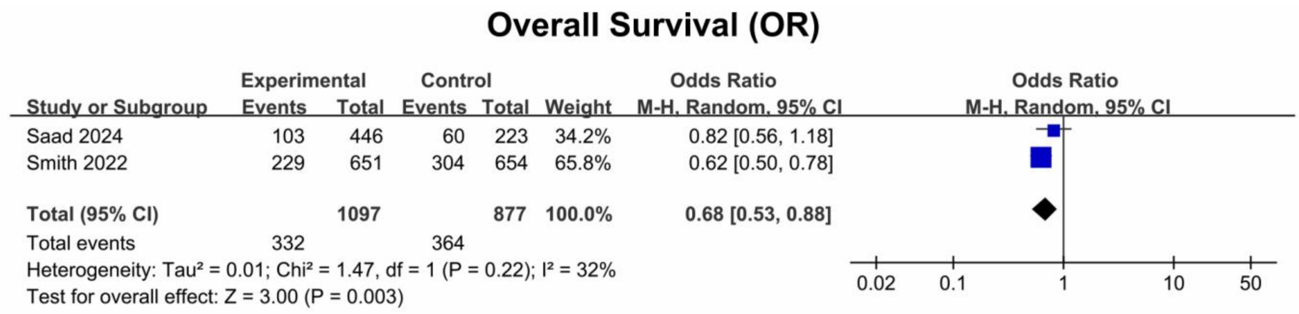

Supplementary Figure S1. Forest plot of overall survival (OS) by odds ratio (OR) for darolutamide versus the control group in the mHSPC cohort. The blue squares represent the OR for each individual study, with the size of the square proportional to the study's weight in the meta-analysis. The horizontal lines indicate the 95% confidence intervals (CIs). The black diamond represents the pooled overall OR, calculated using a random-effects model via the Mantel-Haenszel method. *CI* confidence interval, *mHSPC* metastatic hormone-sensitive prostate cancer, *M-H* Mantel-Haenszel, *OR* odds ratio, *OS* overall survival.

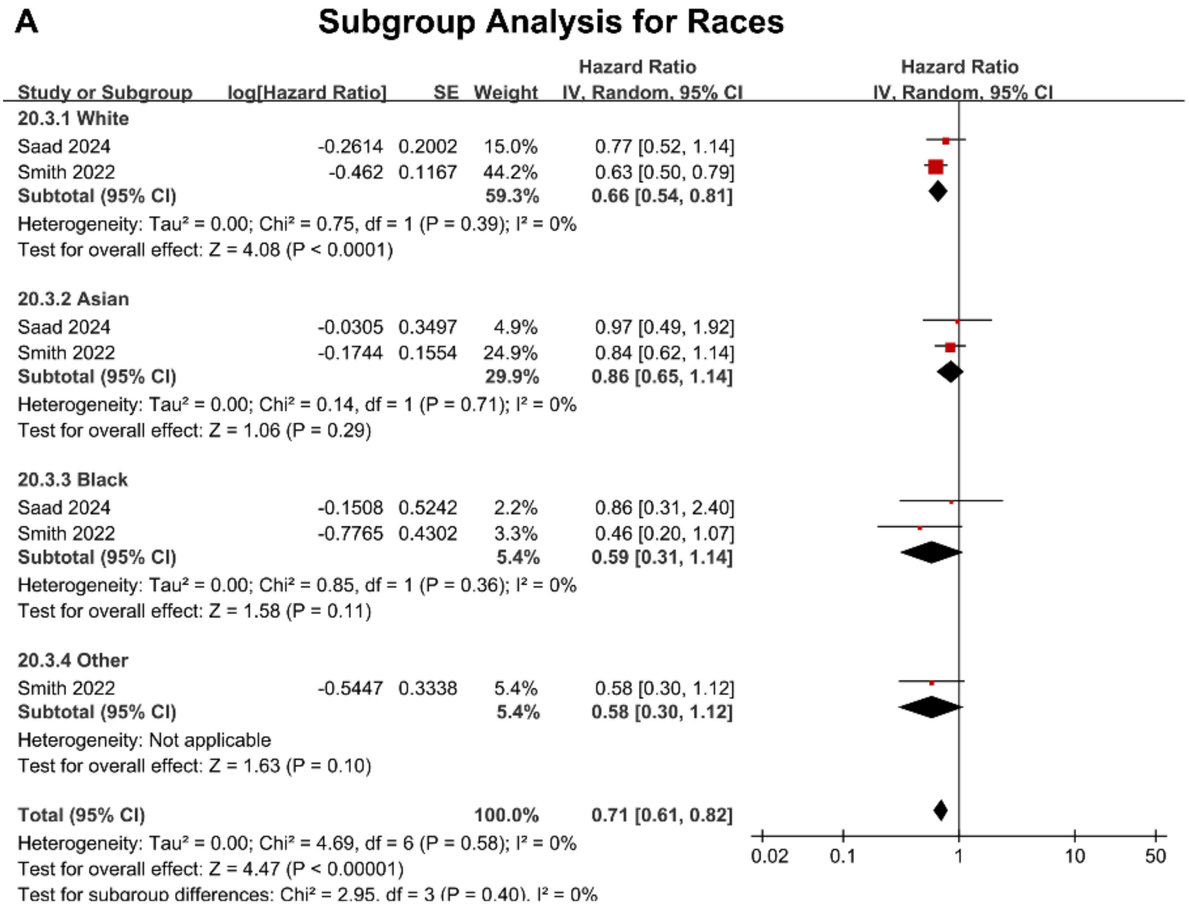

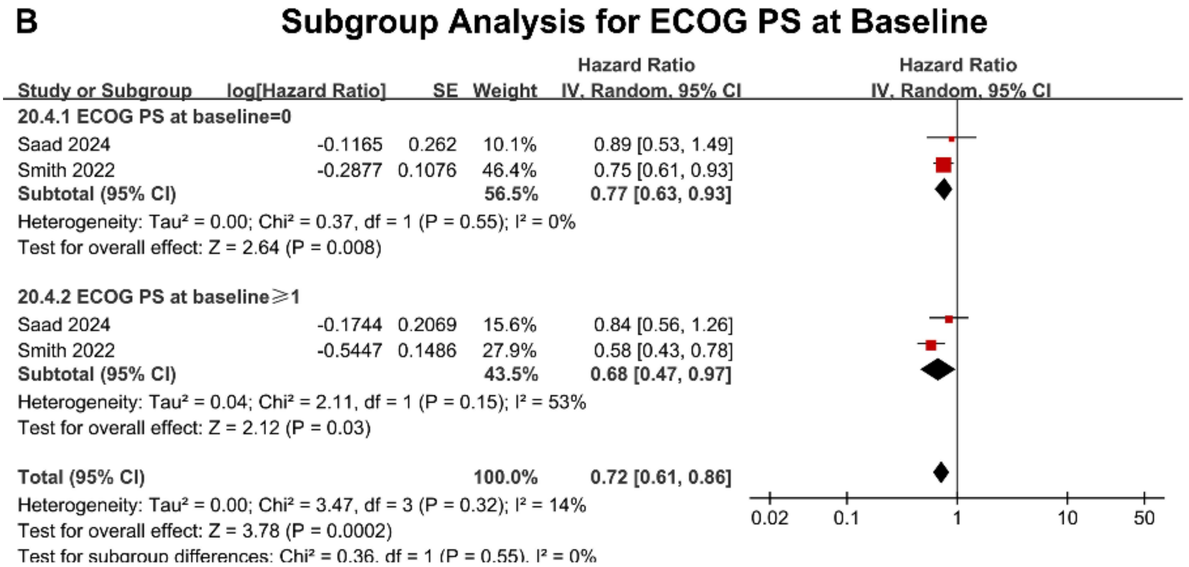

Supplementary Figure S2. Subgroup analyses of overall survival (OS) by hazard ratio (HR) for darolutamide versus the control group in the mHSPC cohort.

(A) The subgroup analysis of treatment effect for races.

(B) The subgroup analysis of treatment effect for ECOG PS at baseline.

The red squares represent the HR for each individual study, with the size of the square proportional to the study's weight in the meta-analysis. The horizontal lines indicate the 95% confidence intervals (CIs). The black diamonds represent the pooled HRs for each subgroup and the overall cohort, calculated using a random-effects model via the generic inverse-variance method. *CI* confidence interval, *ECOG PS* Eastern Cooperative Oncology Group Performance Status, *HR* hazard ratio, *IV* inverse variance, *mHSPC* metastatic hormone-sensitive prostate cancer, *OS* overall survival, *SE* standard error.

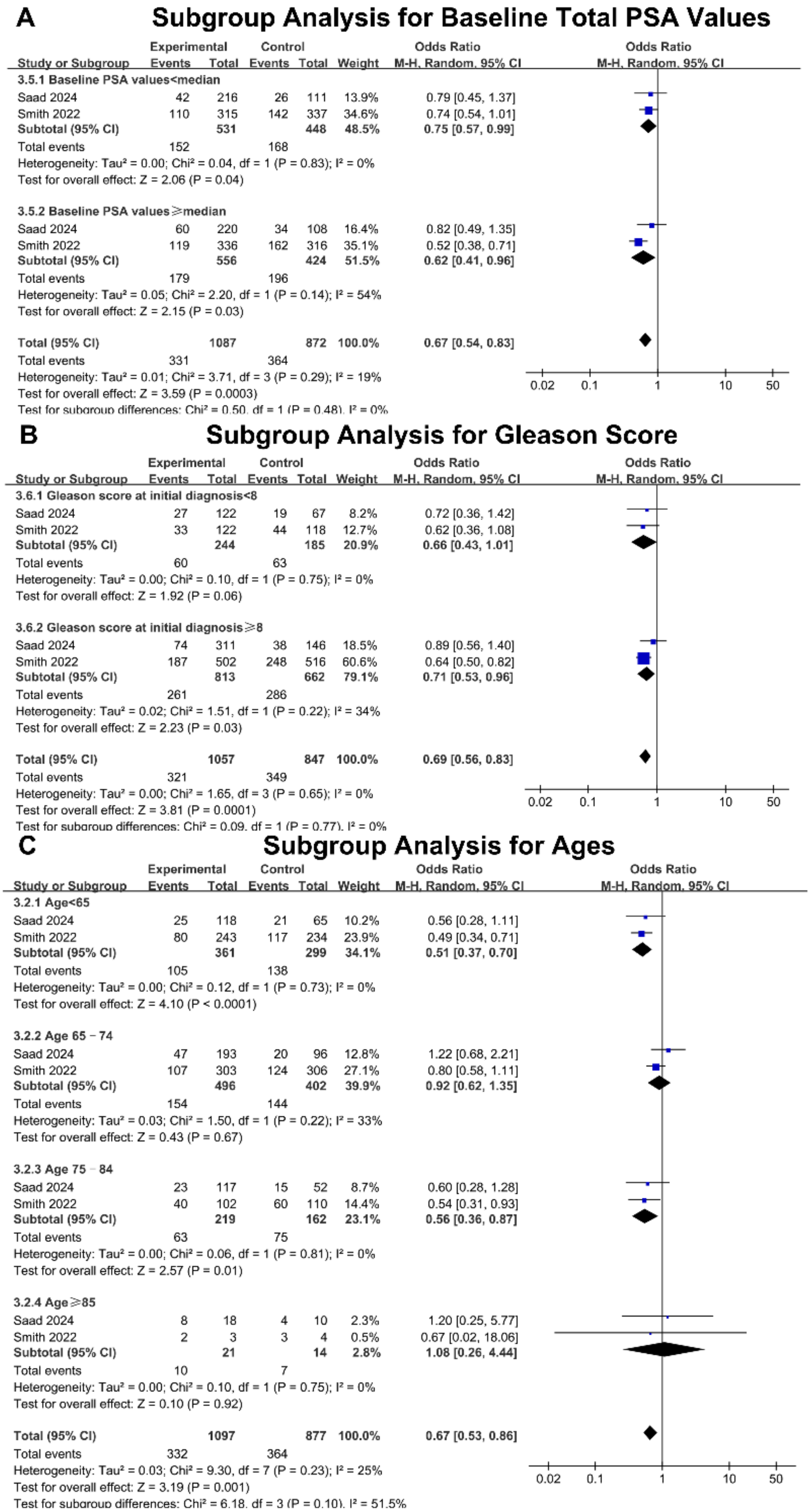

A
Subgroup Analysis for Baseline Total PSA Values
Experimental
Control
Odds Ratio
Odds Ratio
Study or Subgroup
Events
Total
Events
Total
Weight
M-H, Random, 95% CI
M-H, Random, 95% CI
3.5.1 Baseline PSA values<median
Saad 2024 42 216 26 111 13.9% 0.79 [0.45, 1.37]
Smith 2022 110 315 142 337 34.6% 0.74 [0.54, 1.01]
Subtotal (95% CI) 531 448 48.5% 0.75 [0.57, 0.99]
Total events 152 168
Heterogeneity: Tau² = 0.00; Chi² = 0.04, df = 1 (P = 0.83); I² = 0%
Test for overall effect: Z = 2.06 (P = 0.04)
3.5.2 Baseline PSA values≥median
Saad 2024 60 220 34 108 16.4% 0.82 [0.49, 1.35]
Smith 2022 119 336 162 316 35.1% 0.52 [0.38, 0.71]
Subtotal (95% CI) 556 424 51.5% 0.62 [0.41, 0.96]
Total events 179 196
Heterogeneity: Tau² = 0.05; Chi² = 2.20, df = 1 (P = 0.14); I² = 54%
Test for overall effect: Z = 2.15 (P = 0.03)
Total (95% CI) 1087 872 100.0% 0.67 [0.54, 0.83]
Total events 331 364
Heterogeneity: Tau² = 0.01; Chi² = 3.71, df = 3 (P = 0.29); I² = 19%
Test for overall effect: Z = 3.59 (P = 0.0003)
Test for subgroup differences: Chi² = 0.50, df = 1 (P = 0.48), I² = 0%
0.02
0.1
1
10
50
B
Subgroup Analysis for Gleason Score
Experimental
Control
Odds Ratio
Odds Ratio
Study or Subgroup
Events
Total
Events
Total
Weight
M-H, Random, 95% CI
M-H, Random, 95% CI
3.6.1 Gleason score at initial diagnosis<8
Saad 2024 27 122 19 67 8.2% 0.72 [0.36, 1.42]
Smith 2022 33 122 44 118 12.7% 0.62 [0.36, 1.08]
Subtotal (95% CI) 244 185 20.9% 0.66 [0.43, 1.01]
Total events 60 63
Heterogeneity: Tau² = 0.00; Chi² = 0.10, df = 1 (P = 0.75); I² = 0%
Test for overall effect: Z = 1.92 (P = 0.06)
3.6.2 Gleason score at initial diagnosis≥8
Saad 2024 74 311 38 146 18.5% 0.89 [0.56, 1.40]
Smith 2022 187 502 248 516 60.6% 0.64 [0.50, 0.82]
Subtotal (95% CI) 813 662 79.1% 0.71 [0.53, 0.96]
Total events 261 286
Heterogeneity: Tau² = 0.02; Chi² = 1.51, df = 1 (P = 0.22); I² = 34%
Test for overall effect: Z = 2.23 (P = 0.03)
Total (95% CI) 1057 847 100.0% 0.69 [0.56, 0.83]
Total events 321 349
Heterogeneity: Tau² = 0.00; Chi² = 1.65, df = 3 (P = 0.65); I² = 0%
Test for overall effect: Z = 3.81 (P = 0.0001)
Test for subgroup differences: Chi² = 0.09, df = 1 (P = 0.77), I² = 0%
0.02
0.1
1
10
50
C
Subgroup Analysis for Ages
Experimental
Control
Odds Ratio
Odds Ratio
Study or Subgroup
Events
Total
Events
Total
Weight
M-H, Random, 95% CI
M-H, Random, 95% CI
3.2.1 Age<65
Saad 2024 25 118 21 65 10.2% 0.56 [0.28, 1.11]
Smith 2022 80 243 117 234 23.9% 0.49 [0.34, 0.71]
Subtotal (95% CI) 361 299 34.1% 0.51 [0.37, 0.70]
Total events 105 138
Heterogeneity: Tau² = 0.00; Chi² = 0.12, df = 1 (P = 0.73); I² = 0%
Test for overall effect: Z = 4.10 (P < 0.0001)
3.2.2 Age 65－74
Saad 2024 47 193 20 96 12.8% 1.22 [0.68, 2.21]
Smith 2022 107 303 124 306 27.1% 0.80 [0.58, 1.11]
Subtotal (95% CI) 496 402 39.9% 0.92 [0.62, 1.35]
Total events 154 144
Heterogeneity: Tau² = 0.03; Chi² = 1.50, df = 1 (P = 0.22); I² = 33%
Test for overall effect: Z = 0.43 (P = 0.67)
3.2.3 Age 75－84
Saad 2024 23 117 15 52 8.7% 0.60 [0.28, 1.28]
Smith 2022 40 102 60 110 14.4% 0.54 [0.31, 0.93]
Subtotal (95% CI) 219 162 23.1% 0.56 [0.36, 0.87]
Total events 63 75
Heterogeneity: Tau² = 0.00; Chi² = 0.06, df = 1 (P = 0.81); I² = 0%
Test for overall effect: Z = 2.57 (P = 0.01)
3.2.4 Age≥85
Saad 2024 8 18 4 10 2.3% 1.20 [0.25, 5.77]
Smith 2022 2 3 3 4 0.5% 0.67 [0.02, 18.06]
Subtotal (95% CI) 21 14 2.8% 1.08 [0.26, 4.44]
Total events 10 7
Heterogeneity: Tau² = 0.00; Chi² = 0.10, df = 1 (P = 0.75); I² = 0%
Test for overall effect: Z = 0.10 (P = 0.92)
Total (95% CI) 1097 877 100.0% 0.67 [0.53, 0.86]
Total events 332 364
Heterogeneity: Tau² = 0.03; Chi² = 9.30, df = 7 (P = 0.23); I² = 25%
Test for overall effect: Z = 3.19 (P = 0.001)
Test for subgroup differences: Chi² = 6.18, df = 3 (P = 0.10), I² = 51.5%
0.02
0.1
1
10
50

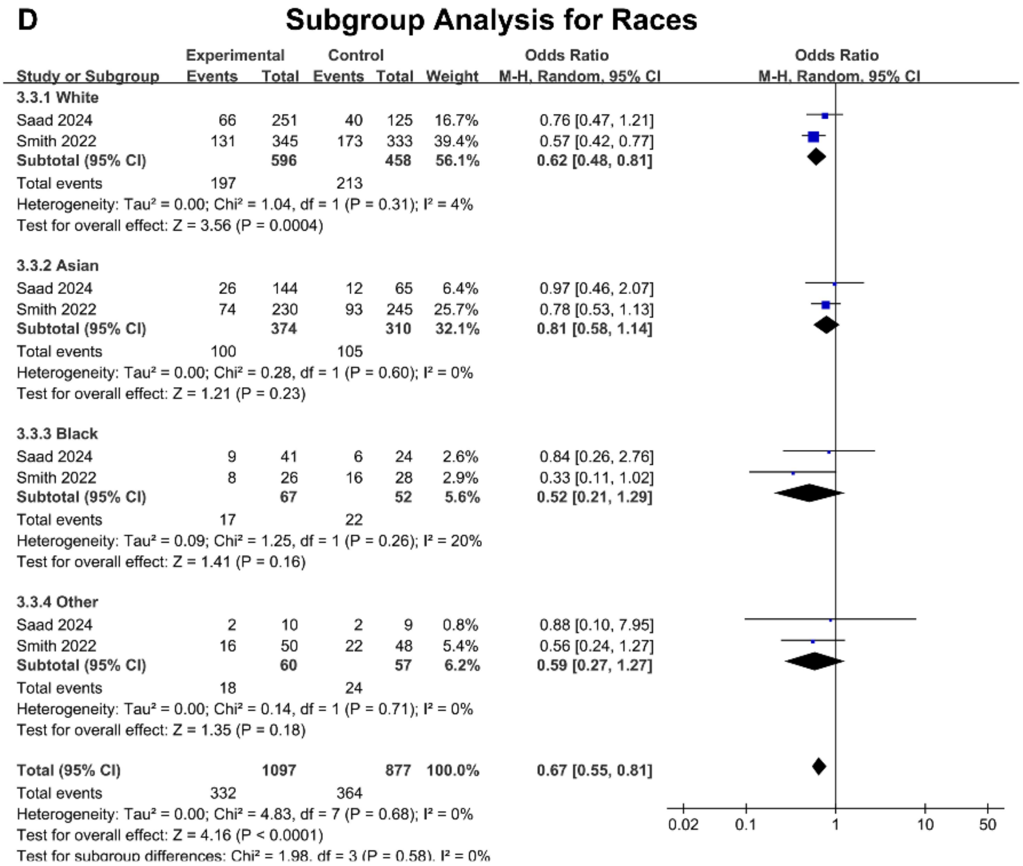

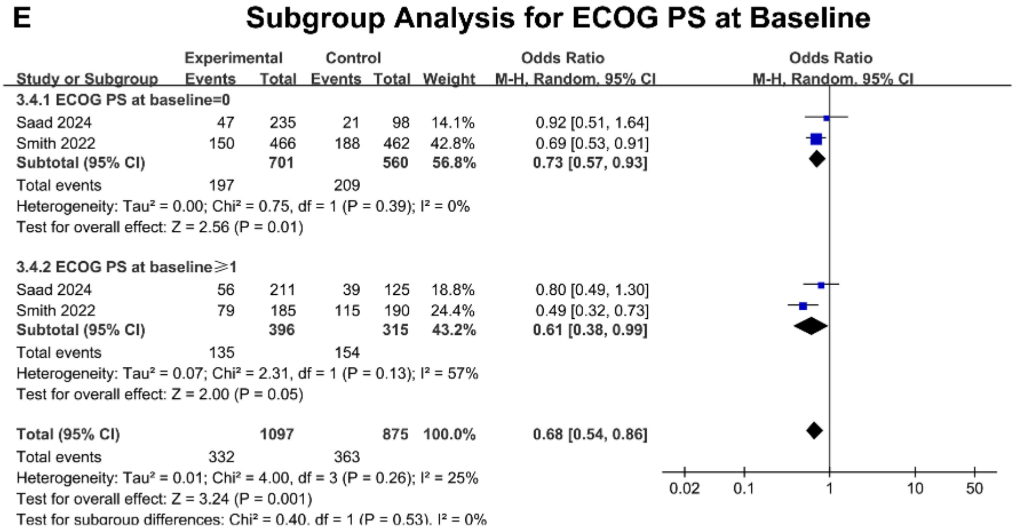

Supplementary Figure S3. Subgroup analyses of overall survival (OS) by odds ratio (OR) for darolutamide versus the control group in the mHSPC cohort.

(A) The subgroup analysis of treatment effect for baseline total prostate-specific antigen (PSA) values.

(B) The subgroup analysis of treatment effect for Gleason score.

(C) The subgroup analysis of treatment effect for ages.

(D) The subgroup analysis of treatment effect for races.

(E) The subgroup analysis of treatment effect for ECOG PS at baseline.

The blue squares represent the OR for each individual study, with the size of the square proportional to the study's weight in the meta-analysis. The horizontal lines indicate the 95% confidence intervals (CIs). The black diamonds represent the pooled ORs for each subgroup and the overall cohort, calculated using a random-effects model via the Mantel-Haenszel method. *CI* confidence interval, *ECOG PS* Eastern Cooperative Oncology Group Performance Status, *mHSPC* metastatic hormone-sensitive prostate cancer, *M-H* Mantel-Haenszel, *OR* odds ratio, *OS* overall survival, *PSA* prostate-specific antigen.

A **Anemia**

Study or Subgroup	Experimental Events	Experimental Total	Control Events	Control Total	Weight	Odds Ratio M-H, Random, 95% CI
Hussain 2023	181	652	163	650	73.9%	1.15 [0.90, 1.47]
Saad 2024	91	445	39	221	26.1%	1.20 [0.79, 1.82]
**Total (95% CI)**		**1097**		**871**	**100.0%**	**1.16 [0.94, 1.44]**
Total events	272		202			

Heterogeneity: Tau² = 0.00; Chi² = 0.03, df = 1 (P = 0.86); I² = 0%
Test for overall effect: Z = 1.38 (P = 0.17)

Odds Ratio M-H, Random, 95% CI

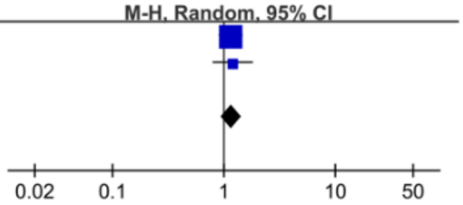

B **Arthralgia**

Study or Subgroup	Experimental Events	Experimental Total	Control Events	Control Total	Weight	Odds Ratio M-H, Random, 95% CI
Saad 2024	55	445	25	221	19.1%	1.11 [0.67, 1.83]
Smith 2022	178	652	174	650	80.9%	1.03 [0.80, 1.31]
**Total (95% CI)**		**1097**		**871**	**100.0%**	**1.04 [0.84, 1.30]**
Total events	233		199			

Heterogeneity: Tau² = 0.00; Chi² = 0.07, df = 1 (P = 0.80); I² = 0%
Test for overall effect: Z = 0.37 (P = 0.71)

Odds Ratio M-H, Random, 95% CI

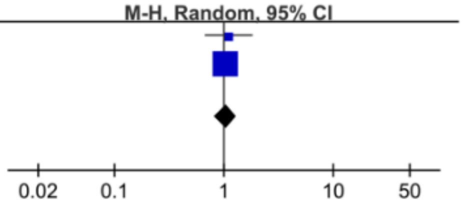

C **Back Pain**

Study or Subgroup	Experimental Events	Experimental Total	Control Events	Control Total	Weight	Odds Ratio M-H, Random, 95% CI
Saad 2024	43	445	23	221	21.3%	0.92 [0.54, 1.57]
Smith 2022	123	652	123	650	78.7%	1.00 [0.75, 1.31]
**Total (95% CI)**		**1097**		**871**	**100.0%**	**0.98 [0.77, 1.25]**
Total events	166		146			

Heterogeneity: Tau² = 0.00; Chi² = 0.07, df = 1 (P = 0.80); I² = 0%
Test for overall effect: Z = 0.16 (P = 0.87)

Odds Ratio M-H, Random, 95% CI

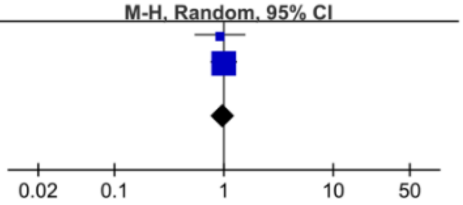

D **Constipation**

Study or Subgroup	Experimental Events	Experimental Total	Control Events	Control Total	Weight	Odds Ratio M-H, Random, 95% CI
Saad 2024	42	445	16	221	16.4%	1.34 [0.73, 2.43]
Smith 2022	147	652	130	650	83.6%	1.16 [0.89, 1.52]
**Total (95% CI)**		**1097**		**871**	**100.0%**	**1.19 [0.93, 1.52]**
Total events	189		146			

Heterogeneity: Tau² = 0.00; Chi² = 0.17, df = 1 (P = 0.68); I² = 0%
Test for overall effect: Z = 1.41 (P = 0.16)

Odds Ratio M-H, Random, 95% CI

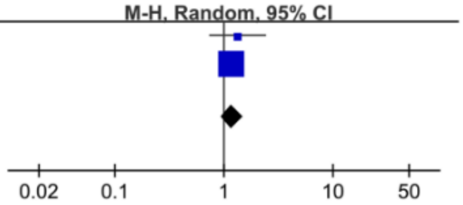

E **Fatigue**

Study or Subgroup	Experimental Events	Experimental Total	Control Events	Control Total	Weight	Odds Ratio M-H, Random, 95% CI
Saad 2024	25	445	18	221	23.3%	0.67 [0.36, 1.26]
Smith 2022	216	652	214	650	76.7%	1.01 [0.80, 1.27]
**Total (95% CI)**		**1097**		**871**	**100.0%**	**0.92 [0.65, 1.29]**
Total events	241		232			

Heterogeneity: Tau² = 0.02; Chi² = 1.42, df = 1 (P = 0.23); I² = 30%
Test for overall effect: Z = 0.50 (P = 0.62)

Odds Ratio M-H, Random, 95% CI

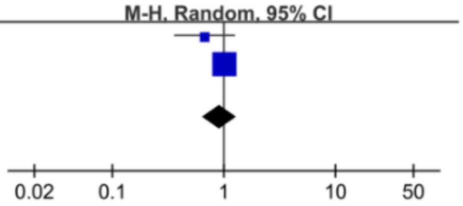

F **Hot Flush**

Study or Subgroup	Experimental Events	Experimental Total	Control Events	Control Total	Weight	Odds Ratio M-H, Random, 95% CI
Saad 2024	41	445	16	221	17.5%	1.30 [0.71, 2.37]
Smith 2022	124	652	122	650	82.5%	1.02 [0.77, 1.34]
**Total (95% CI)**		**1097**		**871**	**100.0%**	**1.06 [0.82, 1.37]**
Total events	165		138			

Heterogeneity: Tau² = 0.00; Chi² = 0.53, df = 1 (P = 0.47); I² = 0%
Test for overall effect: Z = 0.46 (P = 0.64)

Odds Ratio M-H, Random, 95% CI

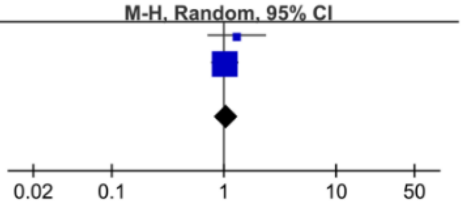

G **Hypertension**

Study or Subgroup	Experimental Events	Experimental Total	Control Events	Control Total	Weight	Odds Ratio M-H, Random, 95% CI
Saad 2024	38	445	19	221	34.2%	0.99 [0.56, 1.77]
Smith 2022	85	652	59	650	65.8%	1.50 [1.06, 2.13]
**Total (95% CI)**		**1097**		**871**	**100.0%**	**1.30 [0.89, 1.92]**
Total events	123		78			

Heterogeneity: Tau² = 0.03; Chi² = 1.45, df = 1 (P = 0.23); I² = 31%
Test for overall effect: Z = 1.35 (P = 0.18)

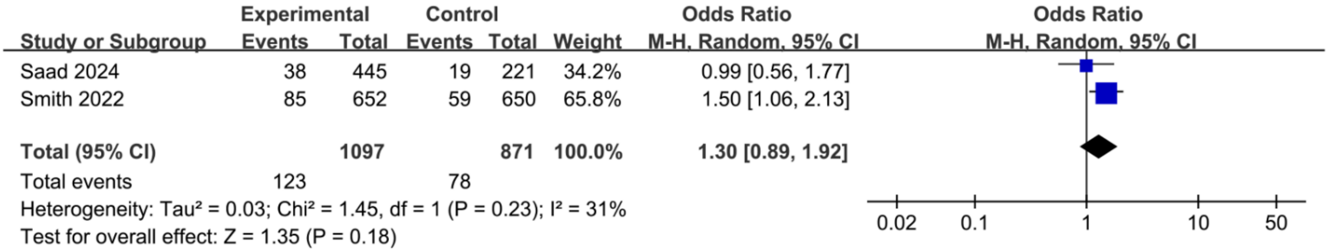

H **Pain in Extremity**

Study or Subgroup	Experimental Events	Experimental Total	Control Events	Control Total	Weight	Odds Ratio M-H, Random, 95% CI
Saad 2024	38	445	20	221	24.1%	0.94 [0.53, 1.65]
Smith 2022	98	652	78	650	75.9%	1.30 [0.94, 1.79]
**Total (95% CI)**		**1097**		**871**	**100.0%**	**1.20 [0.91, 1.59]**
Total events	136		98			

Heterogeneity: Tau² = 0.00; Chi² = 0.95, df = 1 (P = 0.33); I² = 0%
Test for overall effect: Z = 1.28 (P = 0.20)

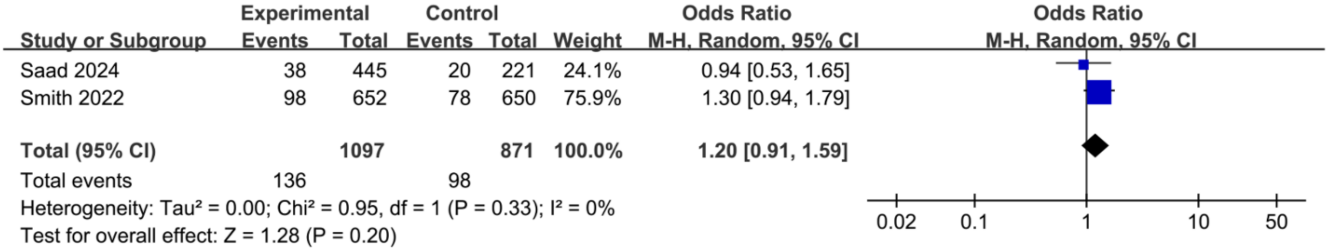

Supplementary Figure S4. Forest plots evaluating specific adverse events by odds ratio (OR) for darolutamide versus the control group in the mHSPC cohort. (A) Anemia. (B) Arthralgia. (C) Back pain. (D) Constipation. (E) Fatigue. (F) Hot flush. (G) Hypertension. (H) Pain in extremity.

The blue squares represent the OR for each individual study, with the size of the square proportional to the study's weight in the meta-analysis. The horizontal lines indicate the 95% confidence intervals (CIs). The black diamonds represent the pooled overall ORs, calculated using a random-effects model via the Mantel-Haenszel method. *CI* confidence interval, *mHSPC* metastatic hormone-sensitive prostate cancer, *M-H* Mantel-Haenszel, *OR* odds ratio.

A Overall Survival

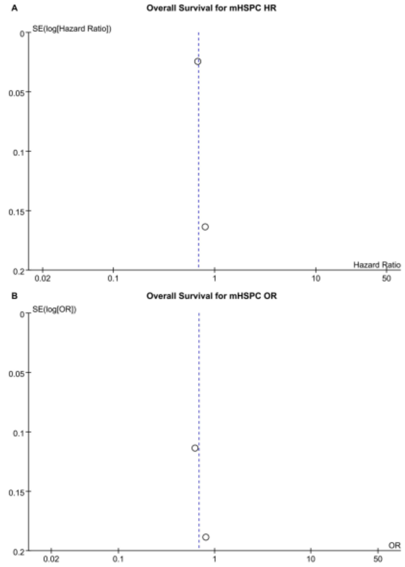

B Specific Adverse Events

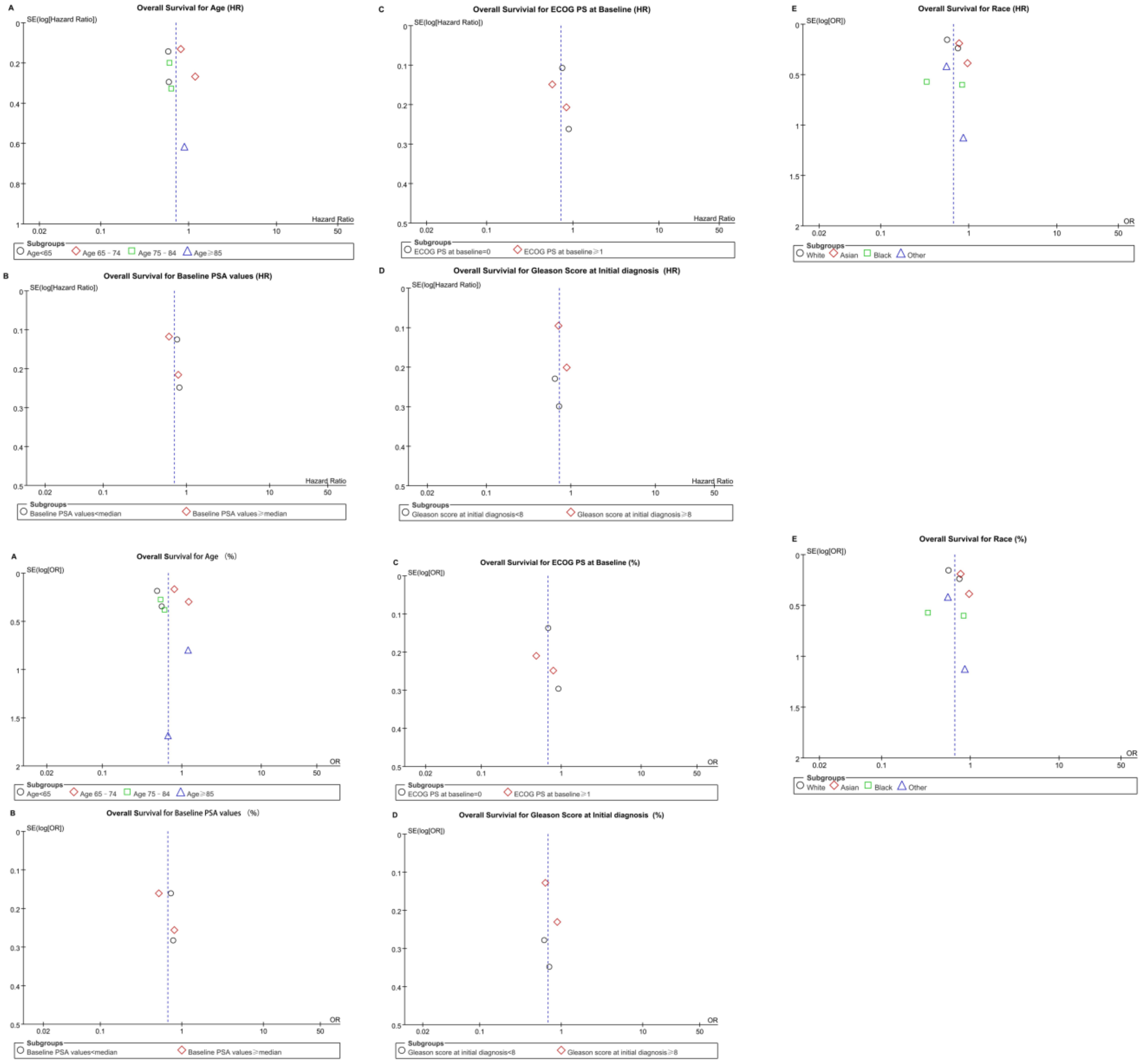

Supplementary Figure S5. Funnel plots assessing potential publication bias for overall survival (OS) in the mHSPC cohort.

(A) Funnel plots for the primary OS analysis.

(B) Funnel plots for the subgroup analyses of OS.

A **Adverse Events**

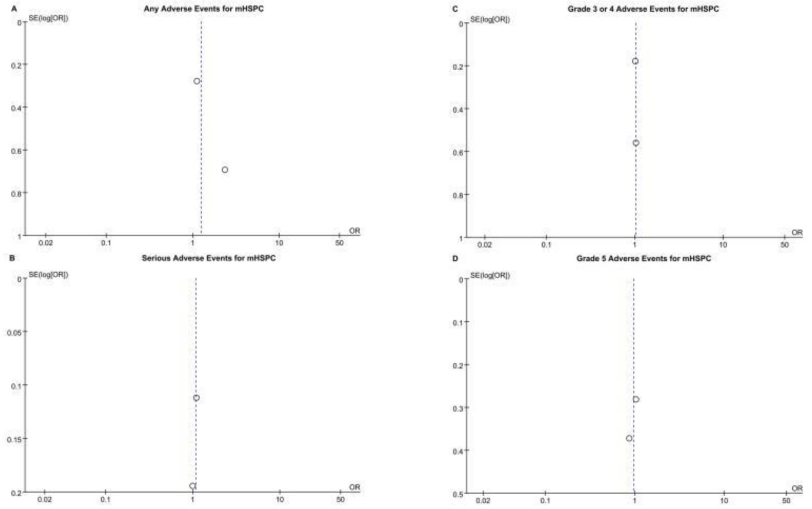

B **Specific Adverse Events**

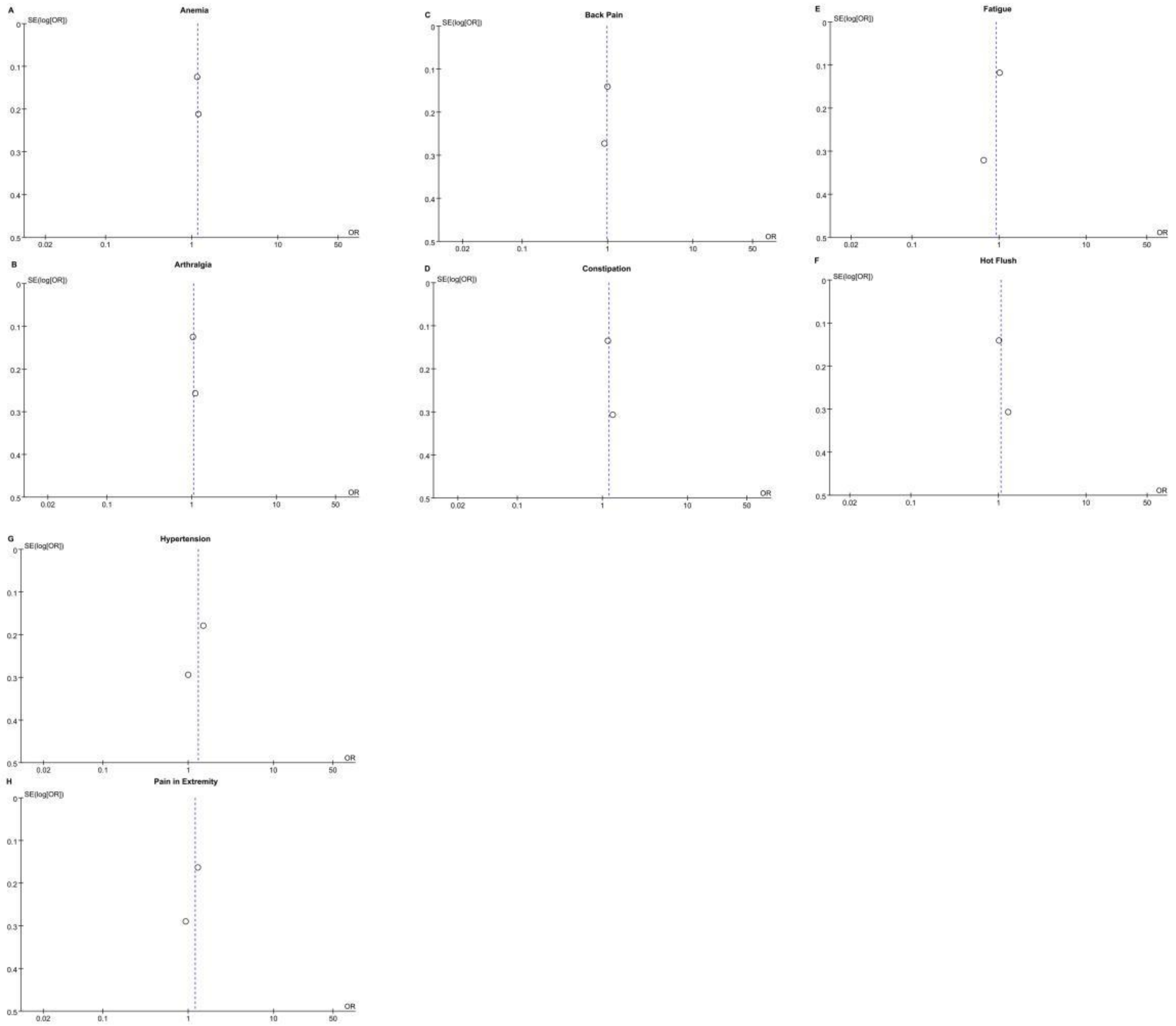

Supplementary Figure S6. Funnel plots assessing potential publication bias for safety outcomes by odds ratio (OR) in the mHSPC cohort.

(A) Funnel plots for adverse events

(B) Funnel plots for specific adverse events.
